# Analysis of Structural Variants Reveal Novel Selective Regions in the Genome of Meishan Pigs by Whole Genome Sequencing

**DOI:** 10.3389/fgene.2021.550676

**Published:** 2021-02-04

**Authors:** Heng Du, Xianrui Zheng, Qiqi Zhao, Zhengzheng Hu, Haifei Wang, Lei Zhou, Jian-Feng Liu

**Affiliations:** ^1^National Engineering Laboratory for Animal Breeding, Key Laboratory of Animal Genetics, Breeding and Reproduction, Ministry of Agriculture, College of Animal Science and Technology, China Agricultural University, Beijing, China; ^2^College of Animal Science and Technology, Yangzhou University, Yangzhou, China

**Keywords:** genome structural variants, whole-genome sequencing, selection signature, reproduction trait, pig

## Abstract

Structural variants (SVs) represent essential forms of genetic variation, and they are associated with various phenotypic traits in a wide range of important livestock species. However, the distribution of SVs in the pig genome has not been fully characterized, and the function of SVs in the economic traits of pig has rarely been studied, especially for most domestic pig breeds. Meishan pig is one of the most famous Chinese domestic pig breeds, with excellent reproductive performance. Here, to explore the genome characters of Meishan pig, we construct an SV map of porcine using whole-genome sequencing data and report 33,698 SVs in 305 individuals of 55 globally distributed pig breeds. We perform selective signature analysis using these SVs, and a number of candidate variants are successfully identified. Especially for the Meishan pig, 64 novel significant selection regions are detected in its genome. A 140-bp deletion in the Indoleamine 2,3-Dioxygenase 2 (*IDO2*) gene, is shown to be associated with reproduction traits in Meishan pig. In addition, we detect two duplications only existing in Meishan pig. Moreover, the two duplications are separately located in cytochrome P450 family 2 subfamily J member 2 (*CYP2J2*) gene and phospholipase A2 group IVA (*PLA2G*4A) gene, which are related to the reproduction trait. Our study provides new insights into the role of selection in SVs' evolution and how SVs contribute to phenotypic variation in pigs.

## Introduction

Structural variants (SVs) are significant contributors to genetic diversity, and it includes insertions/deletions (InDels), inversions, translocations and copy number variations (CNVs) (Alkan et al., 2011; Tattini et al., 2015). SVs can be canonically classified as two types: “balanced” SVs and “unbalanced” SVs (Escaramís et al., 2015; Collins et al., 2017). “Balanced” SVs refer to rearrangements hardly causing major gain or loss of genomic DNA, including inversions and translocations. “Unbalanced” SVs, containing deletions, insertions of novel sequences and duplications, which can result in differences in the numbers of base pairs (bp) among individual genomes (Escaramís et al., 2015; Collins et al., 2017). Genetic variations can contribute to variations in phenotypic traits; previous studies have indicated that SVs were more significantly related to phenotypic diversity than single nucleotide polymorphisms (SNPs) (Chiang et al., 2017). However, SNPs and CNVs are shown to capture 83.6 and 17.7% of total genetic variation in gene expression, respectively (Stranger et al., 2007). Thus, the study of SVs can improve our understanding of the evolution of populations, phenotypic polymorphisms, and the functional genome.

SVs are widespread among different individuals and cell types, such as in non-inherited somatic (Zhuang and Weng, 2015) and germline cells (Guan and Sung, 2016). SVs (>50 bp) can influence large segments of genomes and produce genomic dosage effects (Zhou et al., 2011; Lupski, 2015; Rice and McLysaght, 2017). SV maps of the human, mouse, dog, and many other species have already been successfully constructed (Yalcin et al., 2011, 2012; Berglund et al., 2012; Radke and Lee, 2015; Sudmant et al., 2015). Many genes with SV mutations have also been found to be associated with economic traits in domestic animals. For example, a 110-Kb deletion in the *MIMT1* gene was associated with abortion and stillbirth in cattle (Flisikowski et al., 2010). Similarly, a 176-Kb duplication containing the *PTLR* gene and *SPEF2* gene influenced the growth of chicken feathers (Elferink et al., 2008). The white coat of boars was caused by a 450-Kb duplication involving the *KIT* gene (Giuffra et al., 2002).

SVs have been able to be more precisely detected in pigs with the fast development of genome sequencing. For example, Paudel et al. detected that CNVs might play a role in olfaction, and CNVs were correlated with porcine fatty acid composition and growth traits (Paudel et al., 2015; Revilla et al., 2017). Meanwhile, SVs were discovered in Chinese domestic pigs using whole-genome sequencing (WGS) (Yang et al., 2017). In a previous study, we detected SV regions from a sample size of 13 pigs from Asia and Europe, and found a hotspot region in chromosome X of Asian pig breeds (Zhao et al., 2016). However, for the Meishan pig, as one of the most important Chinese domestic breeds with excellent reproduction features, there is no detailed study of SVs and their functions for this breed.

Here, to investigate the breed-specific SVs in the Meishan pig, we analyzed the SV landscape across the pig genome using WGS data generated from Meishan pig and other 54 pig breeds. Especially, 42 samples (almost 1/8 of all samples) were Meishan pigs to ensure the accuracy of detected SVs in Meishan pigs. We employed a combined detection strategy to detect various types of SVs by running six different software. In this study, we obtained an integrated SV map of 33,698 variants for 305 individuals. Simultaneously, among these variants, 20,770 SVs were detected in the Meishan pig genome. In addition, we identified three critical functional genes correlated with reproduction traits in Meishan pigs. This study provides novel insights into SVs in the pig genome and their potential roles in selective and evolutionary processes in pigs.

## Materials and Methods

### WGS Data Processing

**Figure 1:**
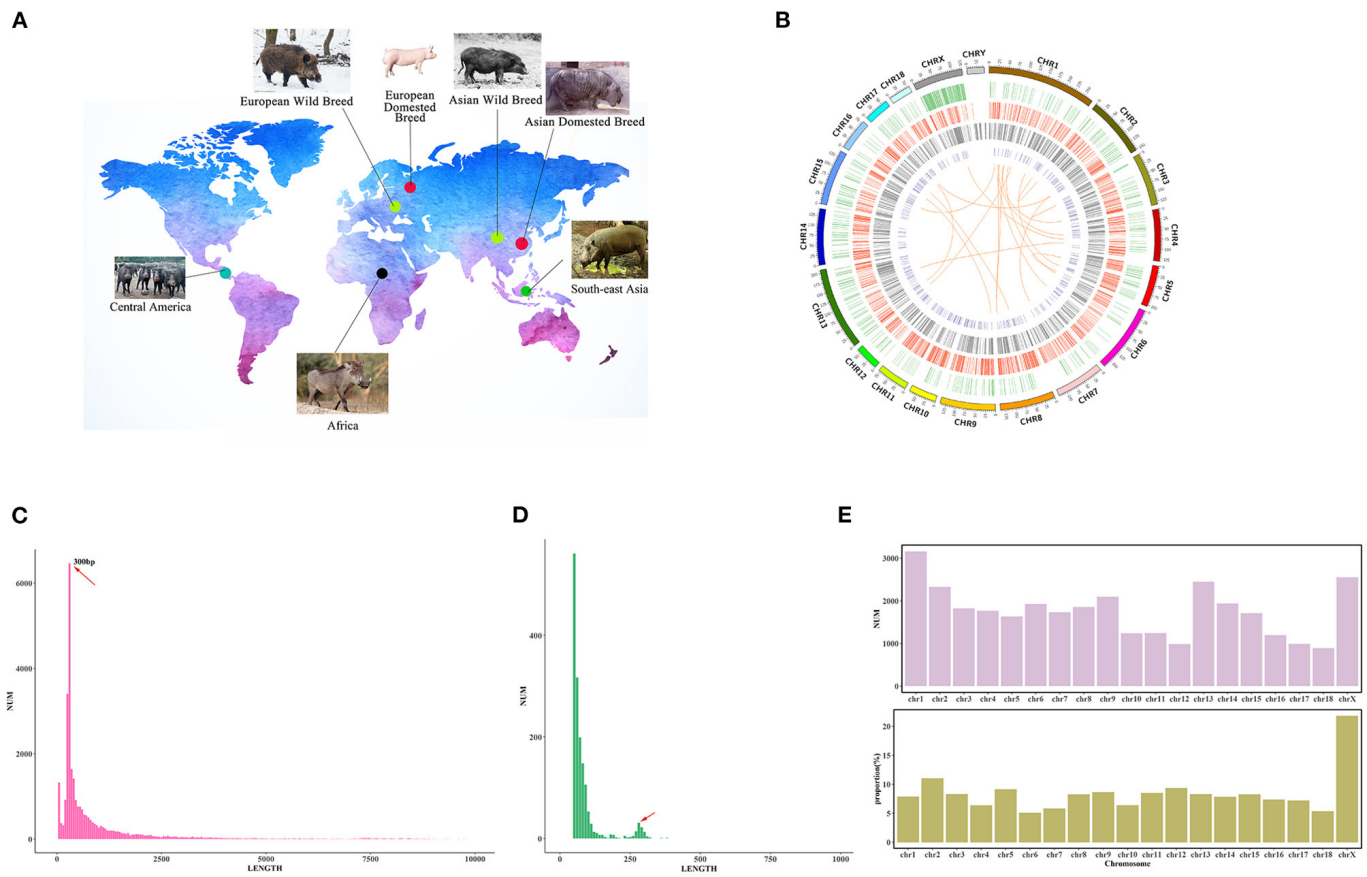
Genome-wide identification of SVs in the pig genome. **(A)** The geographical distribution of breeds used in this study. **(B)** SVs detected from WGS in 305 individuals. Circos plot in which concentric circles show the following (from outer to inner): ideogram of the porcine genome with colored karyotype bands, deletions (>10 kb), insertions, inversions, and CNVs. Circles indicate the location of SVs, and colors indicate the five SV types. **(C)** The size distribution of deletions. The x-axis indicates the length of SVs. The length of SVs is divided into 100 bp bins. The y-axis means the number of SVs which length belongs to the bins. **(D)** The size distribution of insertions. The x-axis indicates the length of SVs. The length of SVs is divided into ten bp bins. The y-axis means the number of SVs which length belongs to the bins. **(E)** SVs distribution in the whole pig genome. The upper figure indicates the number of SVs located in each chromosome. The X-axis indicates the chromosome, and the y-axis means the SV numbers. The figure below shows the proportion of SV regions in each chromosome. The X-axis indicates the chromosome, and the y-axis means the proportion.

To detect SVs in the pig genome, we downloaded WGS data of 305 pigs from NCBI (https://www.ncbi.nlm.nih.gov/) and EBI (http://www.ebi.ac.uk/). These pigs included 55 breeds from Africa, Europe, Asia, South-east Asia, and Central America, and they were further divided into domesticated boars and wild boars (Figure 1A). Detailed information on these pigs listed in Supplementry Table 1. Raw reads were trimmed to a minimum base quality of 20 using NGSQC Toolkit v2.3.3 (Patel and Jain, 2012). Simultaneously, NGSQC Toolkit was also used to remove adapters, low-quality reads (mean quality scores lower than 20), and the reads which were shorter than 70 bp after trimming. The filtered reads were aligned to the *Sus scrofa* 11.1 reference genome using Burrows-Wheeler Aligner v 0.7.17 (Li and Durbin, 2009). Raw mapping results were reordered and sorted by SAMtools v 1.9 (Li et al., 2009) and Picard v1.119. Genome Analysis Toolkit (GATK) v 3.8 (DePristo et al., 2011) was used to correct each individual's alignment results with marking duplicates, local realigning around indels and base quality score recalibration procedures. The read-depth statistics of all downloaded samples were calculated using the DepthOfCoverge function in GATK.

### Whole-Genome Detection of SVs

To enhance SVs' detection accuracy and sensitivity, we used six software involving three computational algorithms (read-pair, read-depth, and split-read methods). These software included Breakdancer v 1.1 (Chen et al., 2009), Pindel v 0.2.5b8 (Ye et al., 2009), DELLY v0.8.1 (Rausch et al., 2012), Manta v 1.5.0 (Chen et al., 2016), Genomestrip v 2.00.1940 (Handsaker et al., 2015), and CNVnator v0.4 (Abyzov et al., 2011).

To control the frequency of false positives and archive a unified pig SV map, we merged the results from all software based on the following consensus principles:

- Merged loci (except for translocations) should show >75% reciprocal overlap among different software;
- The site of SVs was retained when two or more software simultaneously detected the site;
- The start and end positions of SVs were determined using the median method. This median method involved selecting the sites that corresponded to the same SV firstly, and then the median of the start and end positions of the selected sites was calculated as the merged SV's start and end positions.

Lastly, SVs used in downstream analyses were further filtered by rules of deletions (>50 bp, <10 Mb), insertions (>50 bp, <10 Mb), inversions (<10 Mb), duplications (<10 Mb), and translocations (Supplementary Notes).

### Identification of SV Breakpoints

We used a local assembly method to identify SV breakpoints and assess the validity of detected SVs. First, the sequences of candidate SVs in our call set were assembled by falling back to TIGRA-SV v 0.3.7 (Chen et al., 2014). For each site, we used TIGRA-SV to extract the reads and generate local assembly sequences. The sequences around each SV of the reference genome were then used as the reference sequences. Finally, AGE software v0.4 (Abyzov and Gerstein, 2011) was applied to align the assembled sequences of each locus to the corresponding reference and to select validated sites.

### SNP Calling

GATK and SAMtools were used to call SNPs. The Haplotypecaller pipeline of GATK was used to detect and genotype variants. The results of SAMtools were used to select reliable SNP sites from the GATK outputs. The “HardFiltration” function of GATK was then implemented to refine the merged SNP call set (Supplementary Notes).

### Population Genetic Structure and Admixture

To infer the population structure of pigs using detected SVs, we first extracted the genotype predicted by three software (Delly, Manta and Genomestrip), which contained the genotyping process among all six software. The genotype inferred simultaneously by at least two software, was considered as the genotype of a site. All SVs in the SV call set were then genotyped using SVTyper v0.1.4 (Chiang et al., 2015). Next, deletions, SNPs, and SNPs combined with deletions were separately used to estimate the relationships among all populations in our study. Then, we used these data to perform principal component analyses (PCA) (Yang et al., 2011). MEGA-X (Kumar et al., 2018) was employed to construct the phylogenetic tree based on identity-by-Descent (IBD) matrices of the three datasets, respectively. The IBD matrices were calculated by PLINK v1.7(Purcell et al., 2007) using the parameters “–cluster –distance-matrix.” The population structure analysis was conducted by Admixture v1.3 (Alexander et al., 2009). We also estimated the genetic background for each population. Nine possible groupings (K = 2 to K = 10) were calculated by Admixture, and the results were plotted using our R scripts.

### Selection Signature Analysis

Deletions were selected as genetic markers to characterize potential selective signatures in Meishan pigs. In this discovery pipeline, deletions over 100 Kb in length were removed. The absolute allele frequency difference ΔAF between the Meishan population and the other two populations (Duroc and Tibetan wild boars) (ΔAF = abs [AltAF_Meishan_ – mean (AltAF_Duroc_ + AltAF_Tibetan_)]) was calculated in home-written Perl scripts, and it was used to detect selective sweeps in Meishan pigs. Sites in the top 5% of the ΔAF distribution were considered to show signatures of selection. We also used the relative frequency difference (RFD) (Zhou et al., 2015) to detect regions showing selective signatures. The RFD was applied to measure differentiation in the deletions of populations based on variation frequency. The following formula calculated RFD:

$$\begin{array}{l} RFD=\frac{F_{Meishan}-mean\left(F_{Duroc}+F_{Tibetan}\right)}{F_{population}} \end{array}$$

where *F*_*Meishan*_, *F*_*Duroc*_, *F*_*Tibetan*_, and *F*_*population*_ represent the frequencies of deletions in Meishan, Duroc, Tibetan, and the three populations (all samples), respectively. Deletions with low frequencies (<0.01) were removed. Sites in the top 5% of the RFD distribution were considered to be under significant selection. Sites that were claimed as significant both by ΔAF and RFD were added to the selective signature datasets.

### Gene Annotation and Enrichment Analysis

Gene-based and region-based annotations were conducted for all detected SVs in ANNOVAR v 2019Oct24 (Wang et al., 2010). There were 63,041 transcripts (including 13,184 transcripts without coding sequence annotations) for 31,907 unique genes within the Duroc genome used to annotate SVs, and the genes were downloaded from the Ensemble database (*S. scrofa* 11.1). We performed gene enrichment analysis for the annotated genes with KOBAS v3.0 (Mao et al., 2005). We used Fisher's exact test and hypergeometric test as the significance test method. The Benjamini-Hochberg and Benjamini-Yekutieli methods were applied to correct the false discovery rate. Pathways with corrected *P*-values smaller than 0.05 were considered to be significant.

### Identification of Deletions in Genes

Reads aligned to regions with deletions were extracted from each sample's BAM files by bedtools v2.28.0 (https://bedtools.readthedocs.io/). SOAPdenovo2-r241 (Luo et al., 2012) was implemented in assembling the contigs, and the contigs were aligned to the reference sequence by blastn (version 2.9.0+) (ftp://ftp.ncbi.nlm.nih.gov/blast/executables/blast+/LATEST/). The splicing sites were predicted by the Alternative Splice Site Predictor (Wang and Marin, 2006).

### Validation of the SVs by Polymerase Chain Reaction (PCR)

We designed primers using Primer5 (http://www.premierbiosoft.com/primerdesign) for SVs validation (Supplementary Table 2). The DNA templates combined with primers were processed under preheating(93°C, 5 min), amplification (denaturation at 93°C, 40 s, then annealing at 68°C, 30 s, finally elongation at 72°C, 60s, these process executed 33 cycles), and extension(72°C, 7 min) processes. The PCR products from Meishan pig, Tibetan wild boar, and Duroc were separated by size in a 1% agarose gel electrophoresis. SVs were considered successfully validated if their PCR products successfully matched to expected sizes and locations.

## Results

### SV Map Construction of the Pig Genome

We collected WGS data (100-bp read lengths) from 305 pigs of 55 breeds (Li et al., 2013; Ai et al., 2015; Frantz et al., 2015; Zhao et al., 2018) (Supplementary Table 1). After removing low-quality reads, a total of 10,663 Gb of sequencing data with an average 10× mapped read depth was aligned to *S. scrofa* 11.1. We merged six software results to construct an integrated SV map, and the six software contained three detection algorithms: read-pair, read-depth, and split-read algorithms. The high-quality genotyped SVs that we obtained consisted of 30,749 indels (29,173 deletions and 1,576 insertions), 393 inversions, 19 translocations, and 2,537 CNVs (Figure 1B). The comparison of the detected SVs and the public SV database of Ensemble (release 101), showed that 18.43% of SVs identified in our study were new (Supplementary Figure 1). Furthermore, to validate the reliability of the SV map, we applied a local assembly algorithm. We obtained an average alignment rate of 94.08% (Supplementary Figure 2), indicating that our SV map was of high quality. Meanwhile, we randomly validated eight SVs with PCR amplification, and they existed in Meishan pig and Tibetan wild boars, respectively. These eight SVs were successfully confirmed and yielded robust PCR products with the expected sizes in these two breeds, respectively (Supplementary Table 2). In addition, among all the SVs detected in Meishan pig, there were 18,690 indels (17,690 deletions and 1,000 insertions), 112 inversions, 8 translocations, and 1,960 CNVs.

Additionally, we found that the length of all SVs amounted to 212 Mb, which was 8.4% of the total length of the pig genome. Meanwhile, the SV map revealed that CNV occupied the largest proportion of the genome (5.2% of whole genome) among all SV variants, and insertions contributed to the smallest proportion of the genome (0.005% of the genome) (Supplementary Figure 3). These observations demonstrate that the accumulation of repetitive sequences occurred relatively frequently during the pig genome's evolution (Frantz et al., 2016). Interestingly, we found that SVs were not equally occurred in different chromosomes. The proportions of SVs (total length of SVs in the chromosome/total length of the chromosome) in chromosome X (21.84%) and chromosome 2 (11.87%) were higher than other chromosomes (no more than 10%) (Figure 1E). This phenomenon might be correlated to the various haplotype regions in chromosome X of different pig breeds.

The average size for deletions, insertions and inversions were 2,032 bp, 87 bp, and 62,419 bp, respectively. For CNVs, the average size was 50,345 bp. We divided all of the identified SVs into four different length groups (50 bp−1 Kb, 1 Kb−10 Kb, 10 Kb−100 Kb, and 100 Kb−1 Mb). We found that deletions and insertions were enriched in the 50 bp−1 Kb group, while inversions and CNVs were enriched in the 10 Kb−100 Kb group (Supplementary Figure 4). Interestingly, further exploration of the lengths of deletions and insertions in the 50 bp to 1 Kb group revealed that the size of these deletions and insertions tended to be ~300 bp in length (Figures 1C,D). A previous study suggested that the enrichment of indels of the tRNAGlu-derived short interspersed element (SINE/tRNAGlu) contributed to the distribution of indels of this length (Li et al., 2017).

### Annotation of Pig SVs

We used ANNOVAR to annotate all high-confidence SV calls against features in the *S. scrofa* 11.1 annotation. Many SVs were located in intergenic and intronic regions, with 45.81 and 6.56% within 1 kb of a protein-coding gene or long non-coding RNA gene, respectively. 44.37% of all SVs overlapped one or more Ensemble genes. Meanwhile, we also detected SNPs of 305 pigs and compared the annotation results of these SNPs to SVs. Unexpectedly, we found that the distribution of SVs in the pig genome was similar to SNPs' distribution. However, in contrast to SNPs, we found that SVs occurred more frequently in exonic (3.18 vs. 0.78%) and lncRNA exonic regions (1.92 vs. 1.18%) (Supplementary Figures 5, 6). This difference indicates that SVs might be more likely to play a role in altering patterns of gene expression than SNPs.

### Population Genetic Structure Analysis

**Figure 2:**
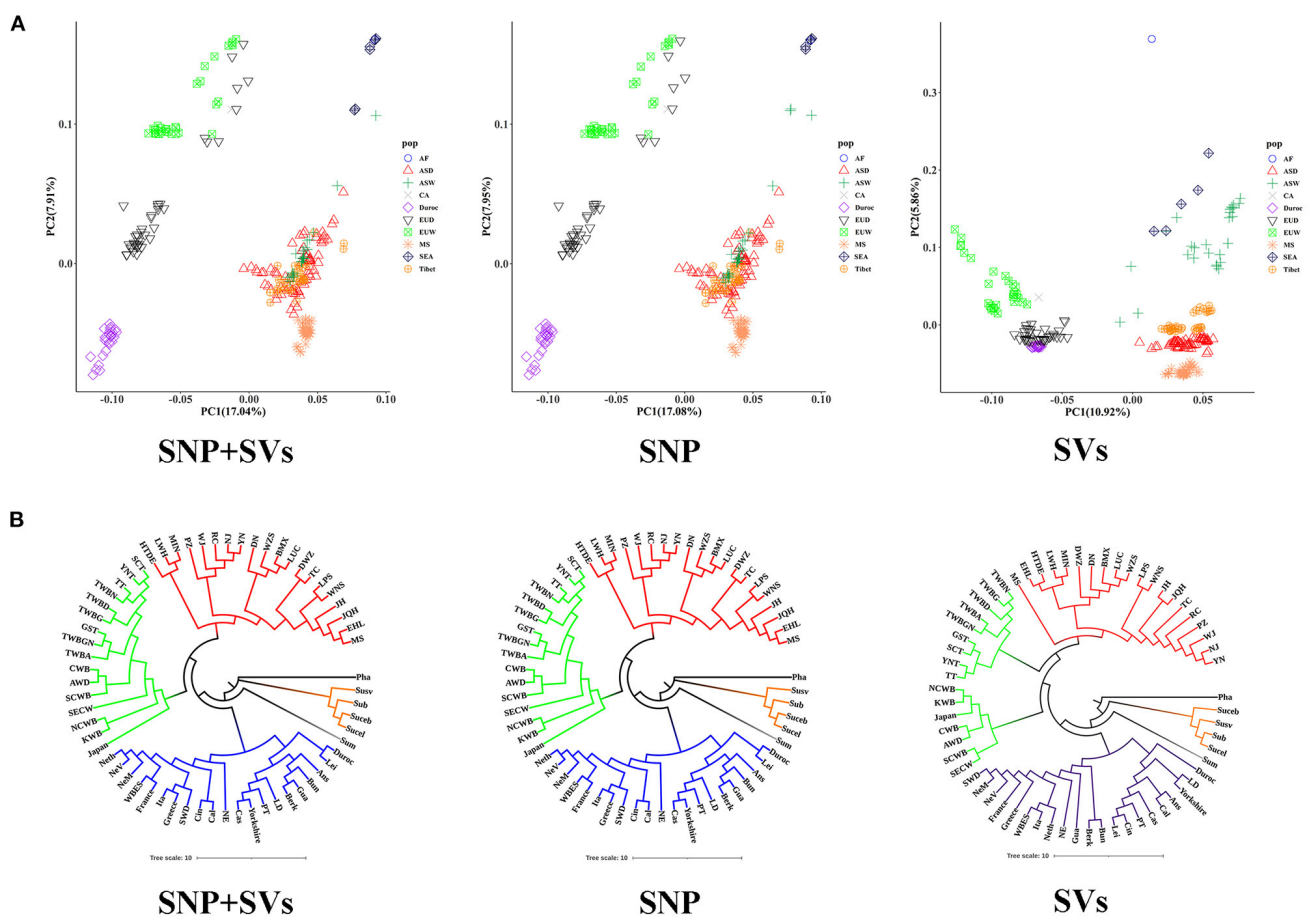
Population diversity and population structure analyses estimated from “SNPs+SVs,” SNP and SVs. **(A)** PCA plot based on “SNPs+SVs”, SNP and SVs. Different colors and shapes represent different pigs from different continents and of different breeds (Meishan pig, Duroc, and Tibetan wild boars are indicated by the other colors and shapes). AF: African boar (*Phacochoerus africanus*), ASD: Asian domestic breed, ASW: Asian wild breed, CA: Central American boars (Guatemala pig), Duroc: Duroc, EUD: European domestic breed, EUW: European wild breed, MS: Meishan pigs, SEA: four populations located in South-east Asia, Tibet: Tibetan wild boars. **(B)** Phylogenetic tree based on “SNPs+SVs”, SNP and SVs (the names of breeds are listed in Supplementary Table 1). Different colors represent different geographic and genetic backgrounds. Black represents African boars. Orange represents breeds from South-east Asia. Gray represents Sumatran pig. Blue represents European breeds. Yellow and red represent Asian wild breeds and Asian domestic breeds, respectively.

We separately used SVs, SNPs, and SNPs combined with SVs (SNPs+SVs) to infer the population genetic structure of all 55 breeds. Because of their more comprehensive genotyping results, deletions were employed as the exclusive representative of SVs in this analysis. Previous studies have demonstrated the effectiveness of using biallelic deletions in population genetic analyses (Sudmant et al., 2015; Bertolotti et al., 2020; Guo et al., 2020). PCA of “SNPs+SVs,” SVs and SNPs all indicated that European populations were clearly distinct from Asian boar populations. A novel finding was that we detected a clear separation between African *Phacochoerus africanus* populations and South-east Asian *Sus* populations (*S. barbatus, S. cebifrons, S. celebensis*, and *S. verrucosus*) by SV-based PCA; however, this separation was not recovered by “SNPs+SVs”-based PCA and SNP-based PCA (Figure 2A). Meanwhile, the above three PCA analyses consistently indicated that Meishan pig clustered with other Asian domestic pigs.

We constructed phylogenetic trees for all populations (Tibetan wild boars were divided into nine subpopulations according to different sampling locations) based on the above “SNPs+SVs,” SVs or SNPs data, respectively. *P. africanus* was used as the outgroup. All populations were divided into four major branches: four breeds from South-east Asia, Sumatran pig, Asian lineages, and European lineages (Figure 2B). Meishan pig was significantly attributed to Asian lineages, and it had close genetic relationships with other Asian domestic breeds.

We then further performed a structure analysis for all the 55 breeds, and the number of presumed ancestral populations (K) was set from 2 to 10. We observed similar patterns when K ranged from 2 to 5 with three different datasets (Supplementary Figures 7–9). In summary, our final set of deletion genotypes captured the expected population genetic structure.

### Analysis of Selective Signals in Meishan Pigs

Svs were known to influence the genome significantly, and they were often associated with specific traits (Sudmant et al., 2015). Our study explored whether the SVs were selected during breed formation and their relationships with specific agricultural traits.

**Figure 3:**
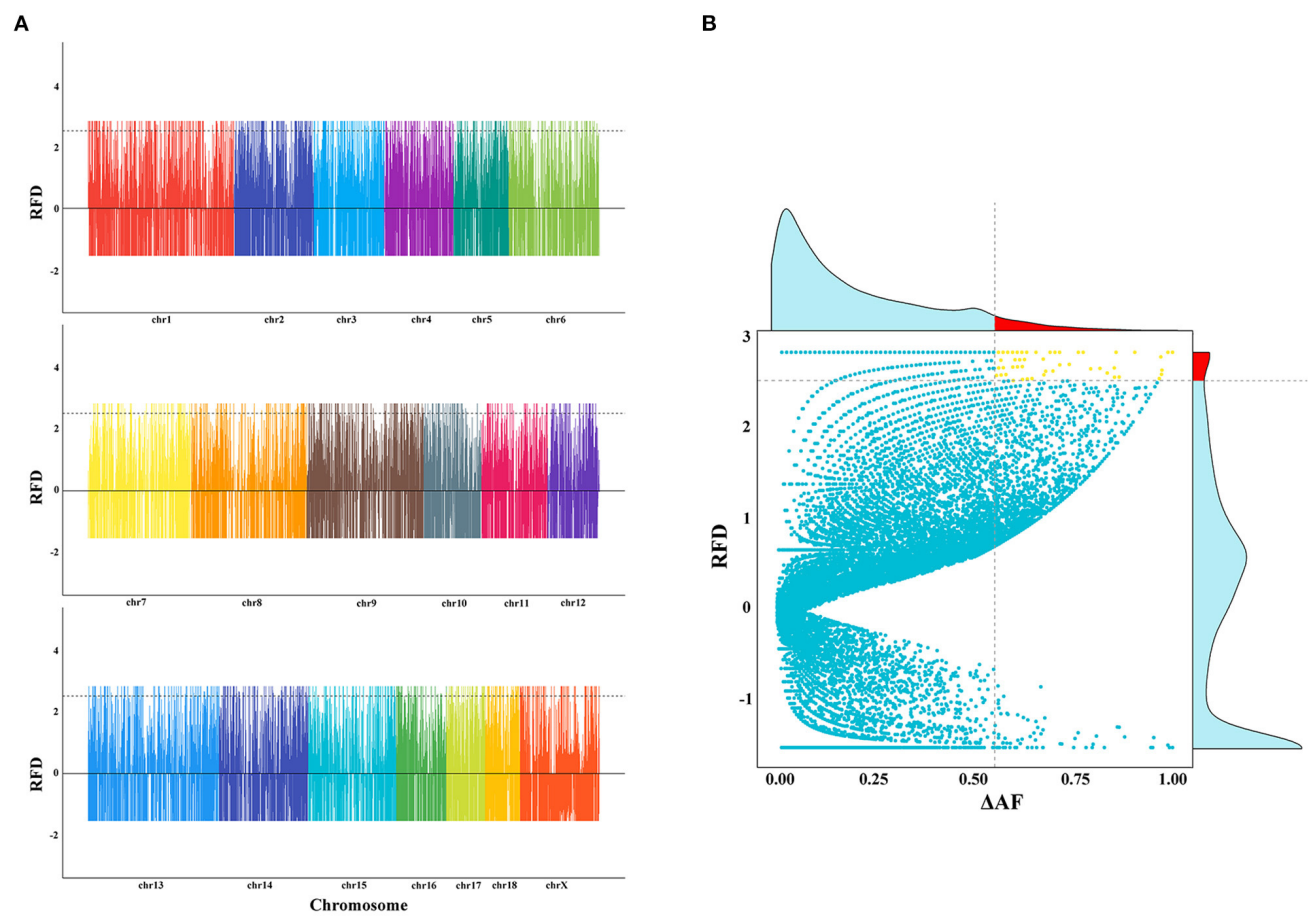
Selective signal analyses using deletion data. **(A)** The plot of RFD values among Meishan, Duroc, and Tibetan wild boar populations. The genome-wide distribution of RFD is calculated using deletion data. The X-axis represents the chromosome. Y-axis indicates the RFD value. **(B)** Definition of selective sweep regions for the Meishan population using deletions. The x-axis corresponds to the ΔAF value, and the y-axis corresponds to the RFD value. The horizontal dashed line and vertical dashed line indicated the top 5% of RFD values and the top 5% of ΔAF values, respectively. The upper-frequency figure means the frequency of ΔAF. The red region indicates the top 5% of ΔAF values. The right frequency figure represents the frequency of RFD. The red region indicated the top 5% of ΔAF values.

**Figure 4:**
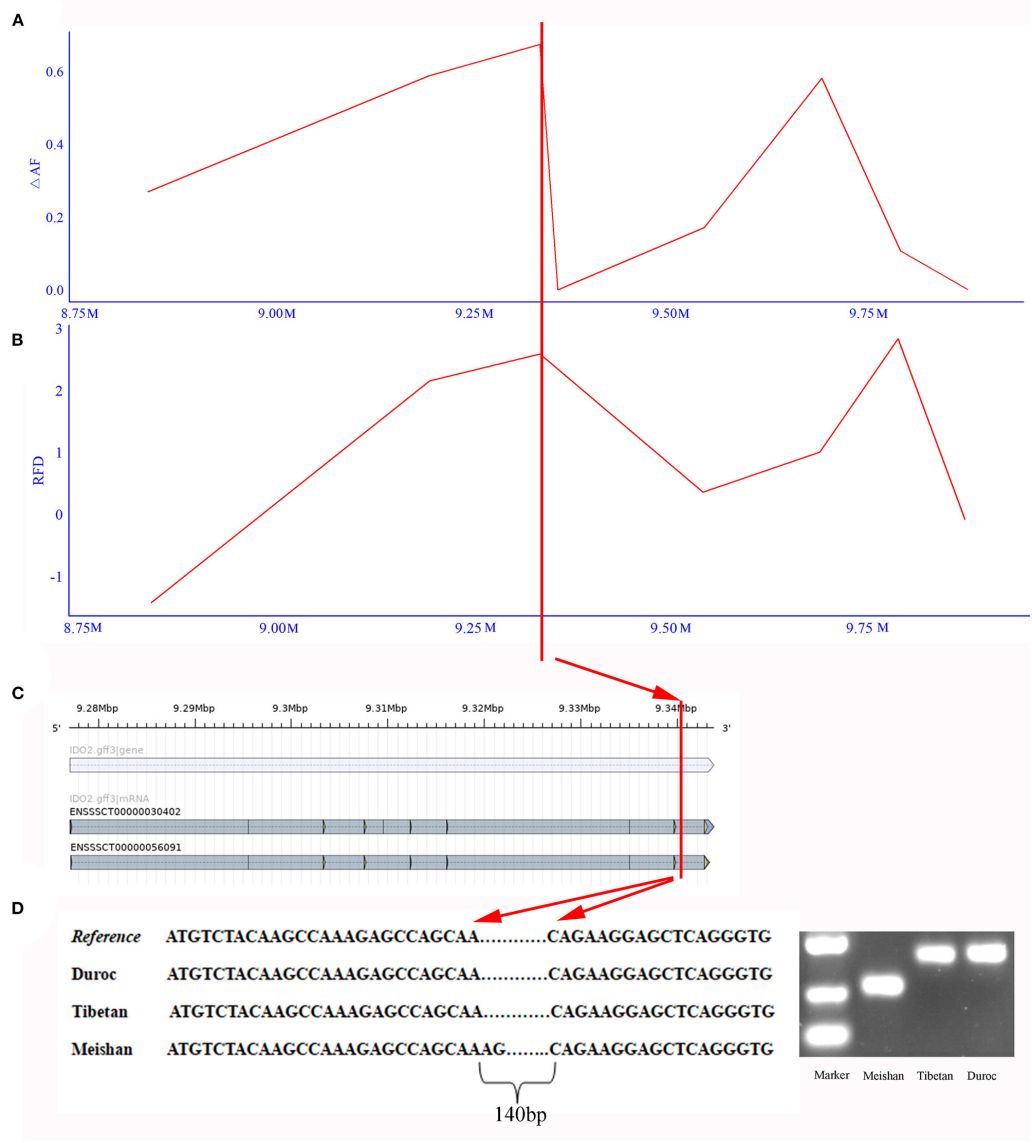
Detected deletion in *IDO2* gene. **(A)** The ΔAF values of deletions around *IDO2* gene. X-axis means the region around the *IDO2* gene in chromosome 17. Y-axis indicates ΔAF value. **(B)** The RFD values of deletions around *IDO2* gene. Y-axis indicates the RFD value. **(C)** The gene structure of *IDO2* gene. The horizontal blue bar represents the gene region, and the horizontal gray bar indicates transcript. **(D)** Deletions in *IDO2* gene of Meishan pig. The left chart indicates the sequence of different breeds in the deleted region. The right chart shows PCR results to detect this deletion in Meishan, Tibetan wild boar, and Duroc breeds.

To test whether SVs experienced positive selection during the breed formation of specific breeds, we further analyzed three breeds, including Meishan, Duroc, and Tibetan wild boars. These three breeds each had their unique traits, like the high reproduction of Meishan pig, cold and low-oxygen tolerant of Tibetan wild boars, and high lean yield of Duroc pig. In this study, we only focused on one trait—high reproduction. We used two different methods to detect SVs under significant selective sweeps. We first used the ΔAF statistic to identify selective signature regions, followed by an RFD test. Only sites discovered by both ΔAF and RFD were considered to be selective signature sites (Figures 3A,B, and Supplementary Figure 10). Finally, a total of 64 deletion regions were found to be under selection (Supplementary Table 3), which covered 37 protein-coding genes. The gene enrichment analysis revealed that these genes were enriched in 16 Gene Ontology pathways (corrected *P* < 0.05, Supplementary Table 4). Among these 37 functionally annotated genes, we identified an important functional gene, *IDO2*, in which a 140-bp deletion occurred in the intronic region only for Meishan pigs (Figures 4A–D).

Furthermore, we predicted the splice sites within the deletion region. This region contained an alternative 5′ splice site and a constitutive 3′ splice site. After removing this deletion, this region appeared two alternative 5′ splice sites and four alternative 3′ splice sites (Supplementary Figure 11 and Supplementary Table 5). Thus, this finding indicated that the alternative splicing and expression level of *IDO2* might be affected by this deletion. *IDO2* played a vital role in maintaining the pregnancy of vertebrate animals (Clark et al., 2005). Simultaneously, dysfunction in *IDO2* could lead to recurrent spontaneous abortion, preeclampsia, preterm labor, and fetal growth restriction (Chang et al., 2018).

### The Role of Other Non-deletion SVs in Meishan Pigs

To identify potential roles in other types of SVs except for deletions, we selected 170 duplications and 77 insertions that specifically existed in Meishan pigs and not in Duroc and Tibetan wild boar populations (Supplementary Tables 6, 7). We annotated these sites to the pig genome to focus on genes that were influenced by these sites.

We detected 84 functional annotated genes, which overlapped with the 170 duplications. Among these genes, we found two genes, cytochrome P450 family 2 subfamily J member 2 (*CYP2J2*) and phospholipase A2 group IVA (*PLA2G*4A), were especially responsible for the ovarian steroidogenesis pathway. This pathway is critical for normal uterine function, the establishment and maintenance of pregnancy, and mammary gland development. Notably, a duplication of 115 kb was observed in the *CYP2J2* only for Meishan pigs (Supplementary Figure 12). Besides, a duplication of length 4,668 bp was detected in *PLA2G4A*, which was only existed in Meishan pig (Supplementary Figure 12). Previous studies have reported that *PLA2G4A* was often up-regulated and contributed to ovarian hormone synthesis (Diouf et al., 2006) and the estrus cycle (Ababneh and Troedsson, 2013). *PLA2G4A* might thus be a potential candidate gene for fertility traits. Therefore, the expression of these two genes was likely affected by the duplicate sequences of these two genes.

## Discussion

Here, we detected SVs across the genome of pigs using six software with NGS data from 55 pig breeds. The SV map identified 6,209 new SVs and greatly contributed to the public SV dataset. To study the relationship of SVs and the specific traits of Meishan pig, we performed selection signature analysis with Meishan pig, Duroc pig and Tibetan wild boar using deletions. Besides, we also discovered the other particular SVs which were only existed in Meishan pig and further predicted their functions. Finally, we identified critical functional genes associated with SVs related to the reproduction traits of pigs. This research provides new insights into the role of selection in the evolution of SVs and candidate genes for porcine fertility traits.

With the population genetic structure analysis results using “SNP+SVs,” SVs and SNPs, we observed that SVs could capture the expected genetic structure. This finding indicated that SVs, especially for deletions, had the power to discriminate divergences among distantly related lineages. Therefore, SVs might also be considered in future studies of genetic structure analysis. In recent years, SV markers have become a promising and useful tool for forensic identification (Caputo et al., 2017) and biogeographic research (Levy-Sakin et al., 2019). Previous studies have successfully used SVs to characterize the genetic diversities and genetic relationships in many species, like human groups (Xie et al., 2018), peach accessions (Guo et al., 2020), and salmon groups (Bertolotti et al., 2020). These findings also demonstrated the high power of SVs in population analyses. Thus, our results provided a preliminary framework for how to apply SVs in genetic structure analysis.

Our study discovered that 44.37% of all SVs overlapped one or more Ensemble genes, slightly higher than SNPs (43.07%). Additionally, because of long base pairs for each SV, exonic regions were covered more frequently by SVs than SNPs. This indicated that SVs might have more tight relationships with traits. A previous study showed that candidate variants associated with peach fruit traits were identified in genome-wide association studies (GWAS) using SVs (Guo et al., 2020). Meanwhile, Song et al. demonstrated that presence and absence variations-GWAS (PAV-GWAS) complement SNP-GWAS in identifying associations to traits (Song et al., 2020). SVs deserve further research in variants association analysis for different traits.

One of the consequences of SVs being targets of selection was differences in SV frequencies among populations (Zhou et al., 2015). Thus, analysis of variation in the frequencies of SVs among different populations might promote the identification of important genomic regions under selection and functional genes in these regions. Compared with our previous study examining selective signals in Meishan pigs using SNPs (Zhao et al., 2018), we obtained 64 non-overlapping selective signature regions and identified a potential functional gene, *IDO2*, associated with pig fertility. Previous studies have shown that *IDO2* played a vital role in the maintenance of pregnancy in vertebrate animals (Moraes et al., 2018). The intronic variation could alter the expression of proteins by influencing the alternative splicing of relevant genes (Xu et al., 2018). We found a loss of a small segment in *IDO2*, which affected its alternative splicing. We speculated that this loss might influence the expression level of *IDO2* and affected fertility traits in Meishan pigs. DNA methylation was linked to the regulation of *IDO2* expression and altered patterns of *IDO2* expression; however, DNA methylation can also adversely affect the outcomes of pregnancy (Spinelli et al., 2019). Thus, *IDO2* could be further targeted for the future epigenomic study of porcine fertility.

We also investigated important functional genes strongly correlated with other traits of Meishan pigs covered by the SVs except for deletions. For instance, we found that a duplication influenced the *PLA2G4A* gene. Previous studies have shown that this gene was associated with premature ovarian failure (POF), which could cause amenorrhoea, infertility, and early onset of menostasis (Kuang et al., 2014). In the pig industry, POF syndrome often reduces the productive lifespan of sows and can cause substantial economic losses. Thus, *PLA2G4A* could be targeted in future studies of POF in both pigs and humans. Overall, our findings contributed to further understanding and exploring the genome characteristics of Meishan pig and other pig breeds.

## Data Availability Statement

The datasets presented in this study can be found in online repositories. The names of the repository/repositories and accession number(s) can be found in the article/Supplementary Material.

## Author Contributions

J-FL conceived and designed the experiments. HD performed all analysis processes and wrote the manuscript. XZ and QZ performed PCR validation of structural variation. J-FL, ZH, HW, and LZ revised the paper. All authors read and approved the final manuscript.

## Conflict of Interest

The authors declare that the research was conducted in the absence of any commercial or financial relationships that could be construed as a potential conflict of interest.
