# Supplementary material for: Analysis of Structural Variants Reveal Novel Selective Regions in the Genome of Meishan Pigs by Whole Genome Sequencing
Source: Front Genet. 2021 Feb 4;12:550676. doi: 10.3389/fgene.2021.550676 (PMC7890942; doi:10.3389/fgene.2021.550676)
Supplement: Supplementary file 1 [file Data_Sheet_1.PDF]

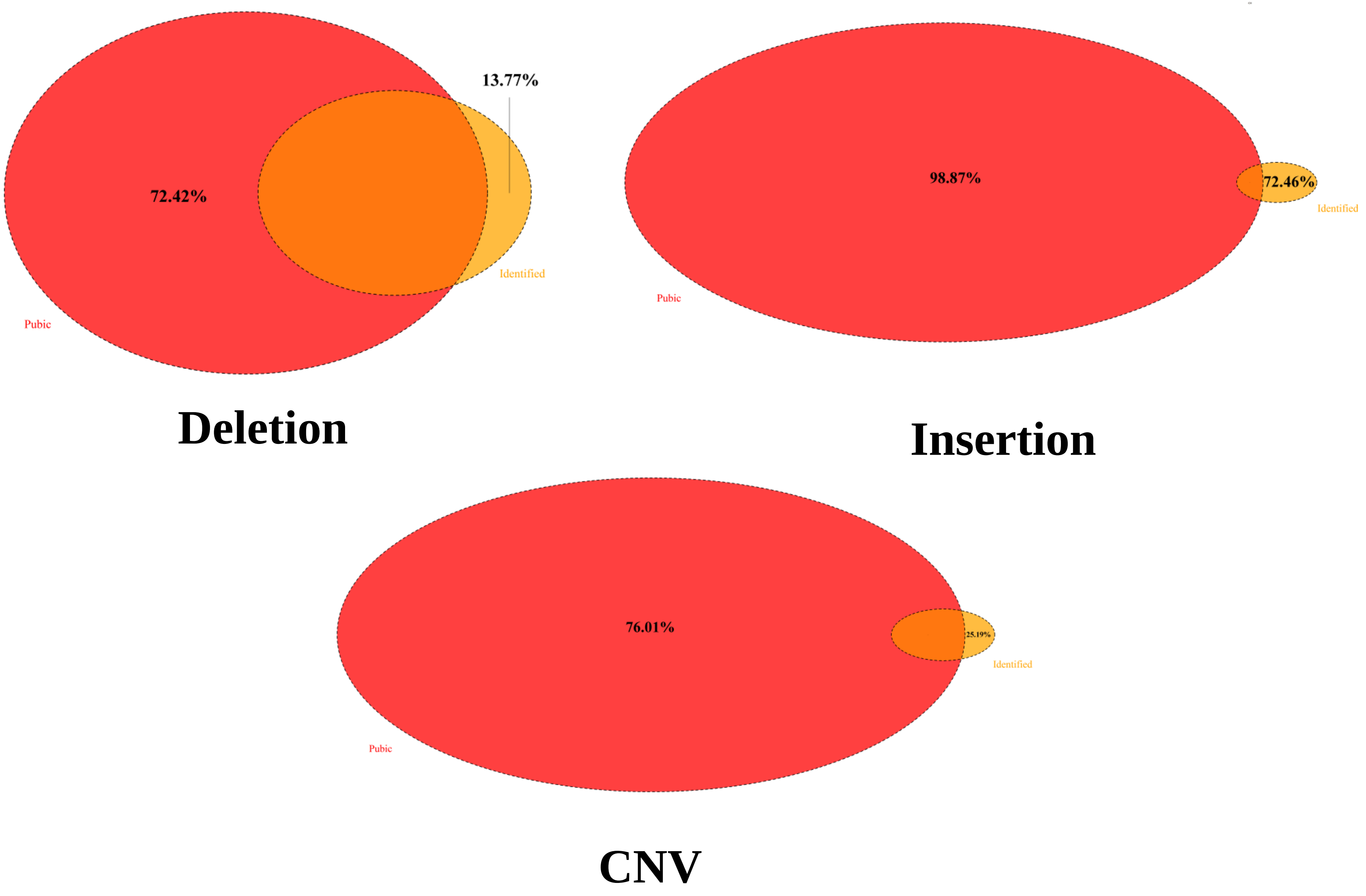

Figure S1 The detected SVs overlap with public SV dataset.

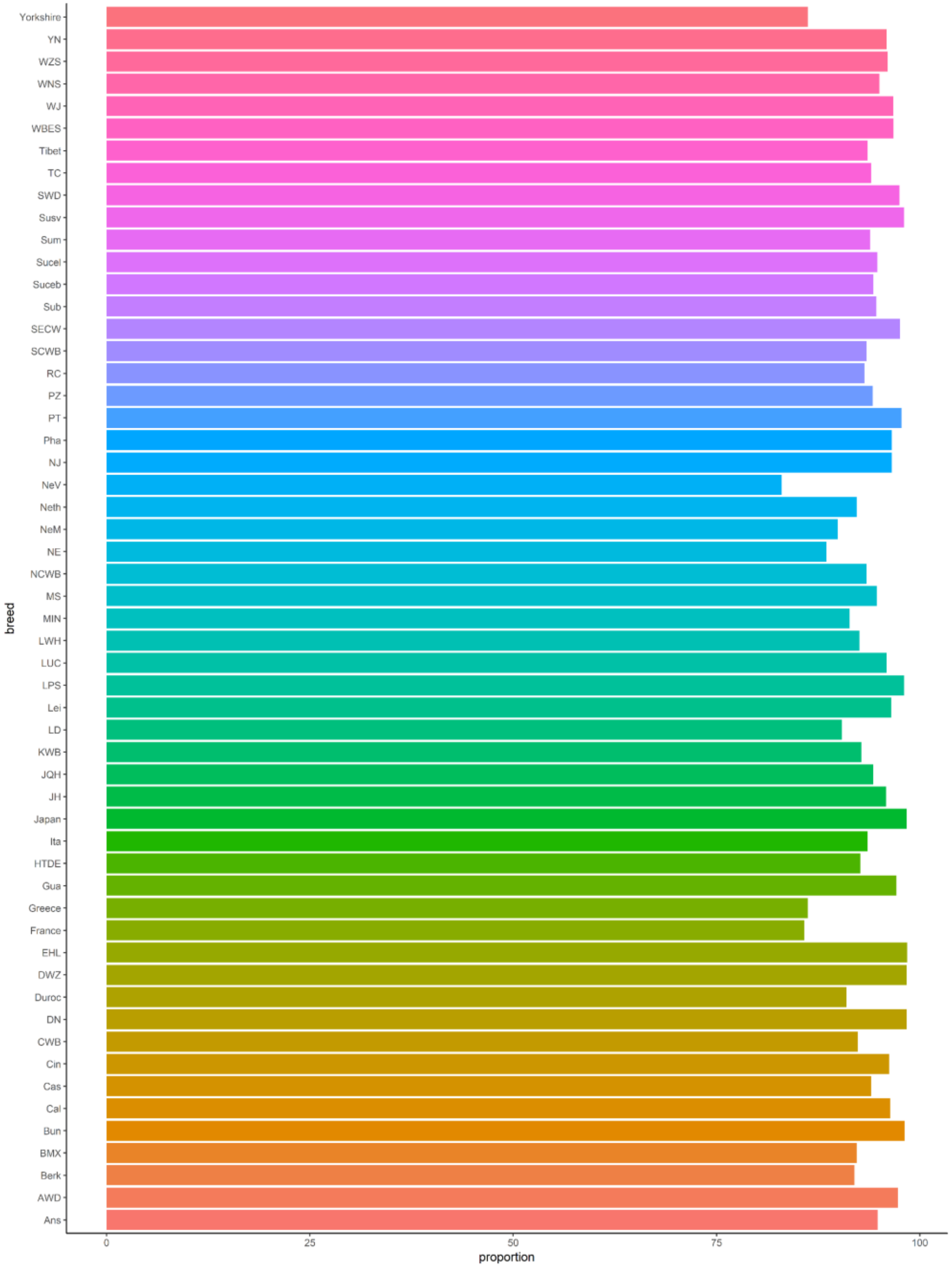

Figure S2 The local alignment ratio in different breeds.

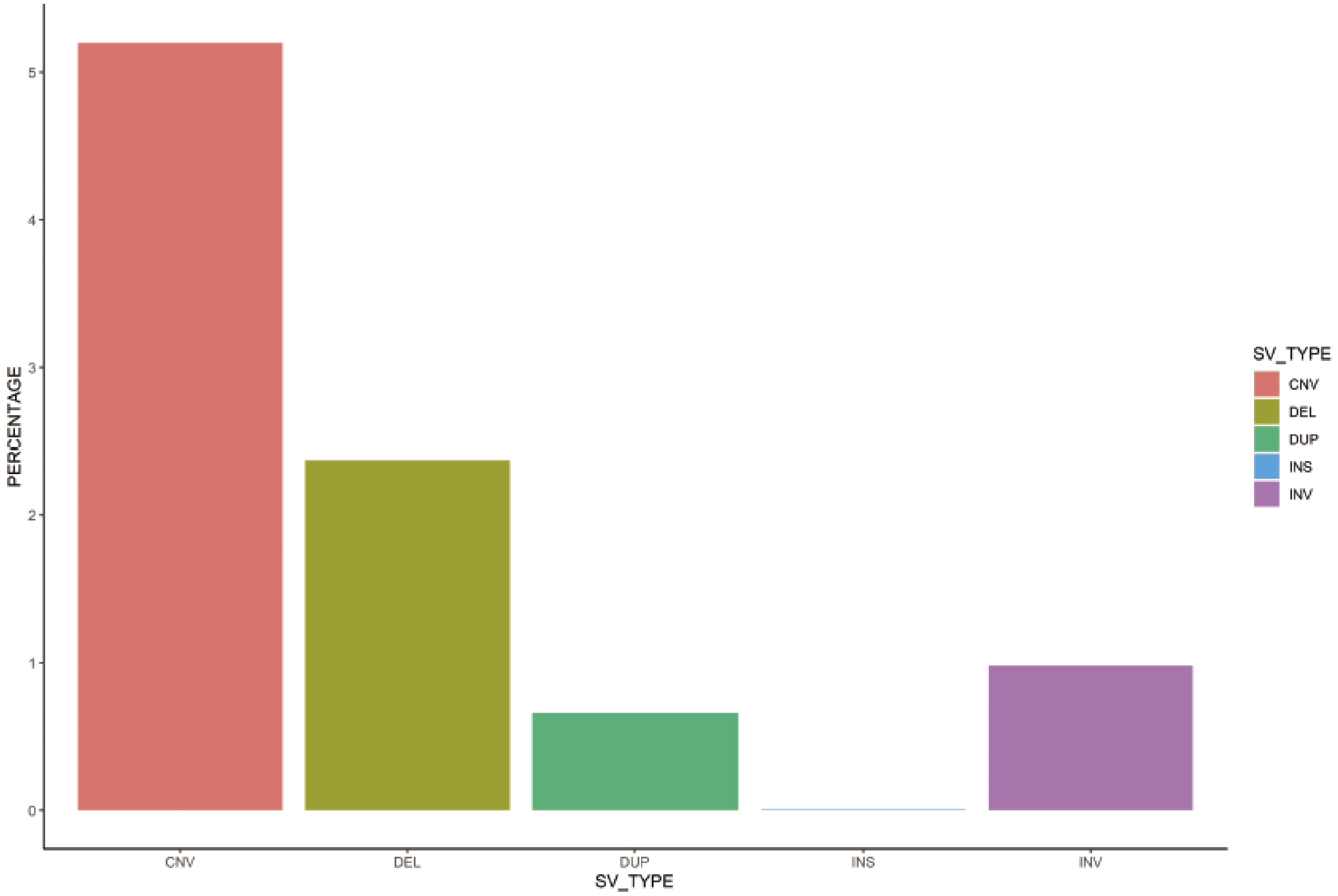

Figure S3 The proportion of different SV types occupied the pig genome.

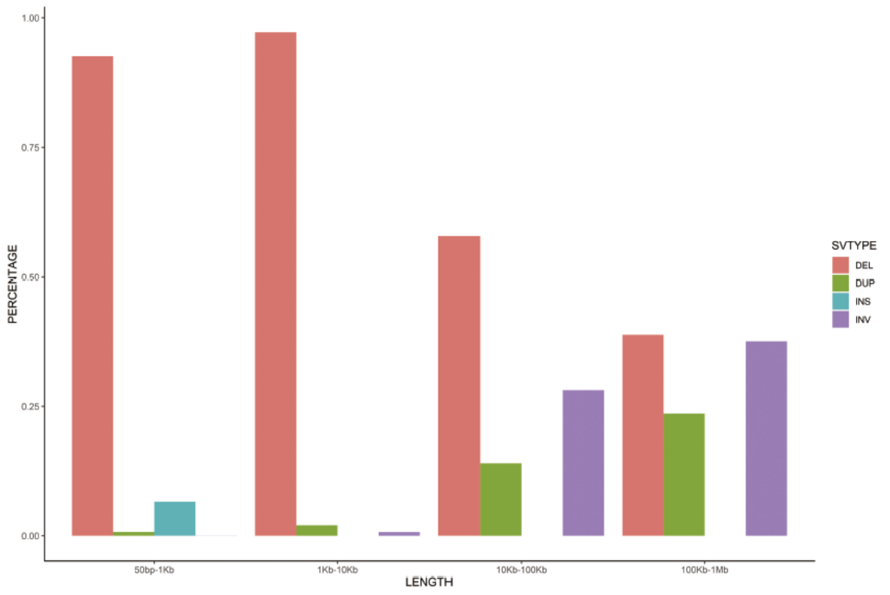

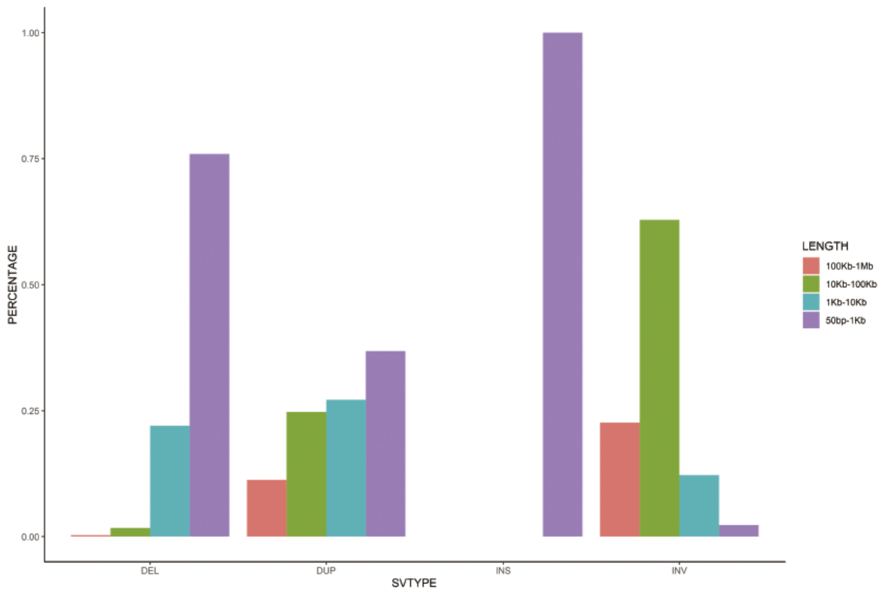

Figure S4 The length of different SVs.

The upper figure showed that the percentage of the length of one sv type belonged to which levels when divided the lengths of SVs into four levels. The lower figure showed that in one sv type, the percentage of four lengths' levels respectively

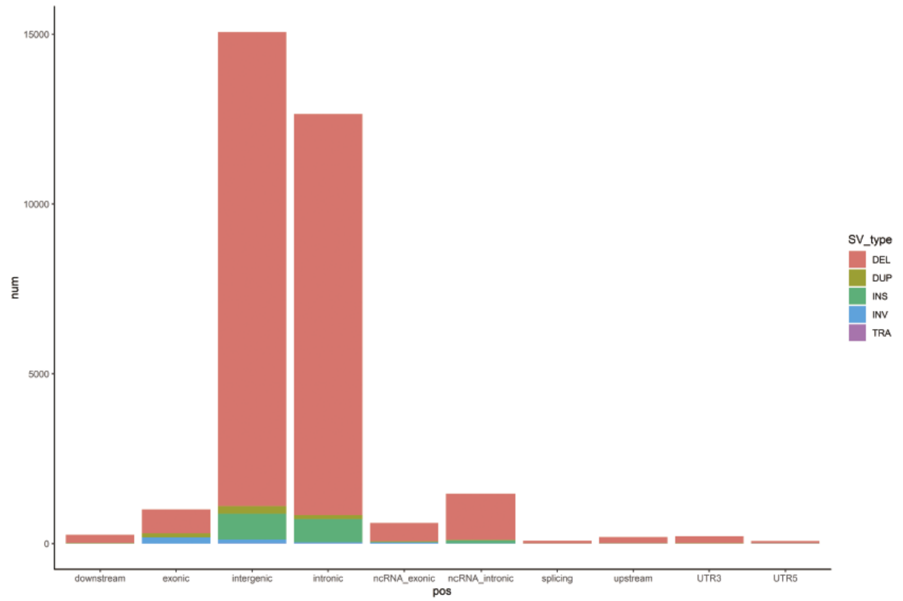

Figure S5 The proportion of different SV types annotated in different regions of pig genome.

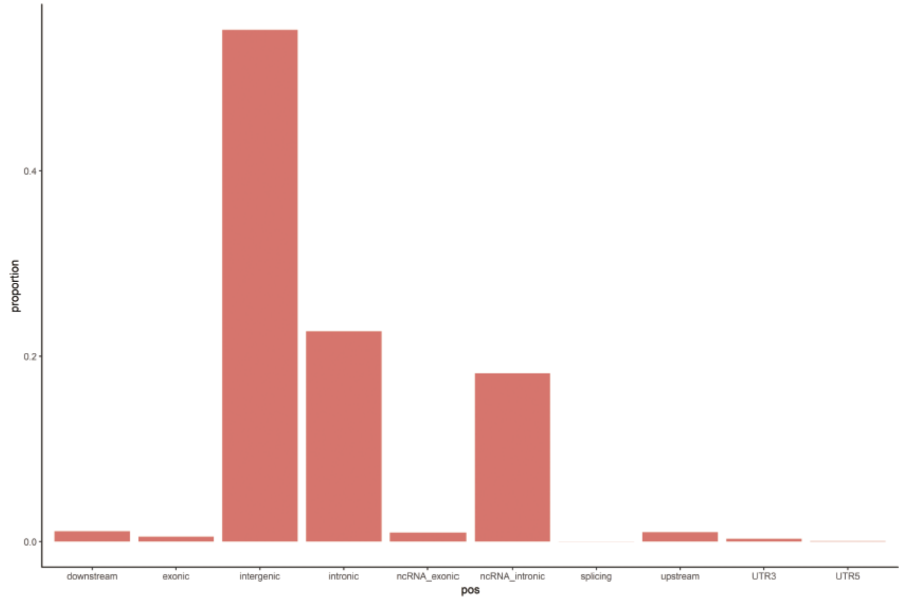

Figure S6 The proportion of SNPs annotated in different regions of pig genome.

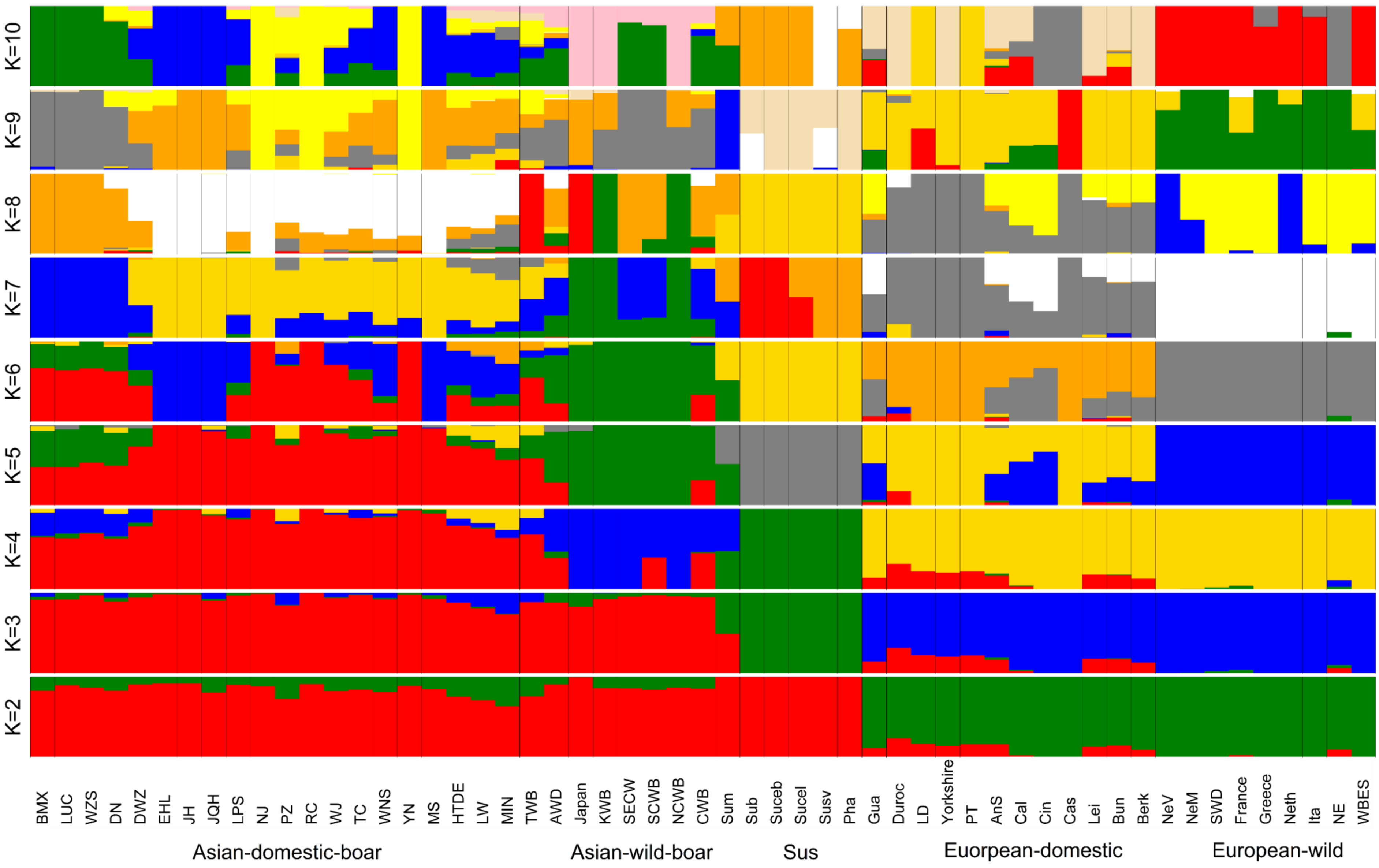

Figure S7 Admixture results of “SNPs+SVs”.

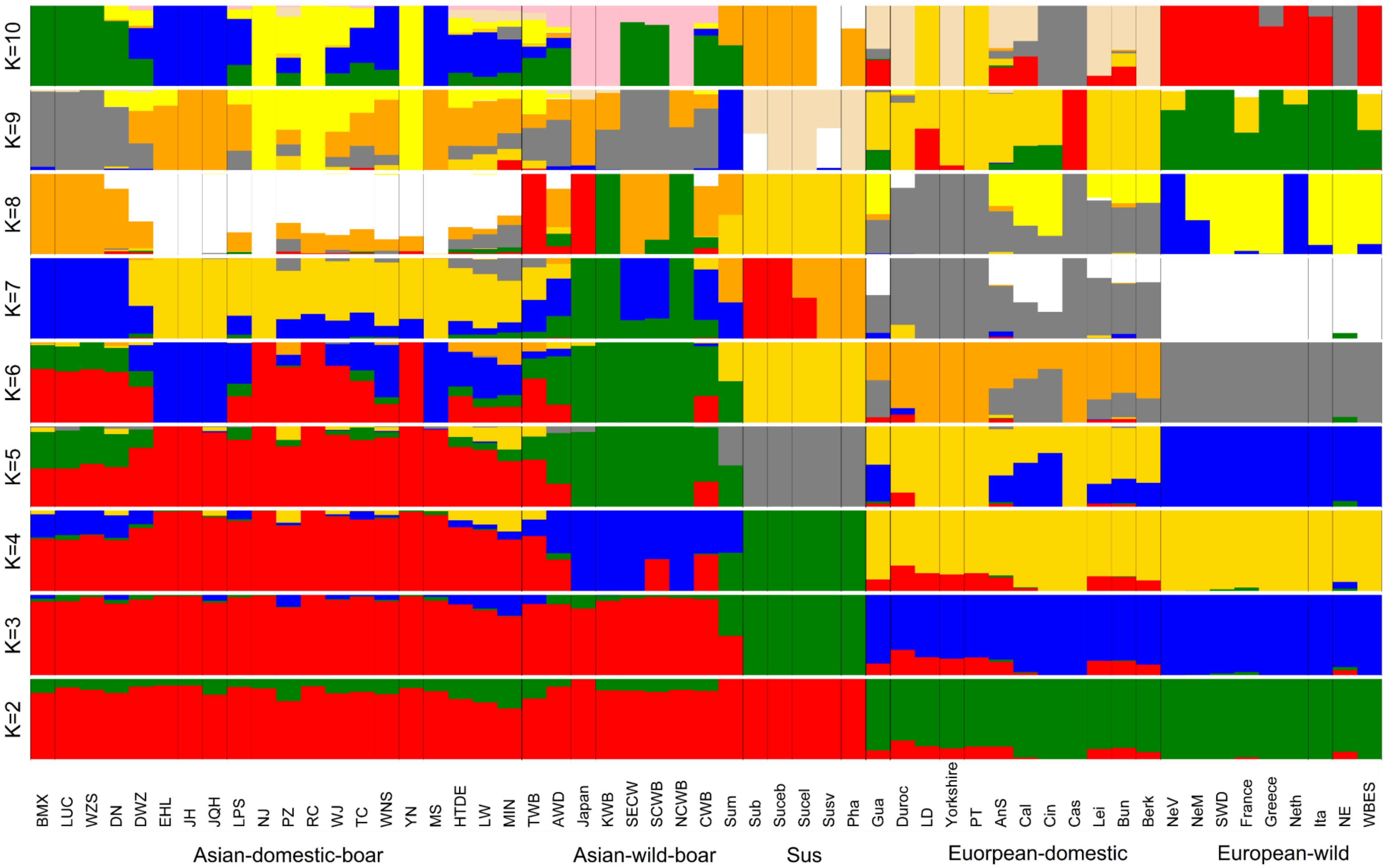

Figure S8 Admixture results of SNPs.

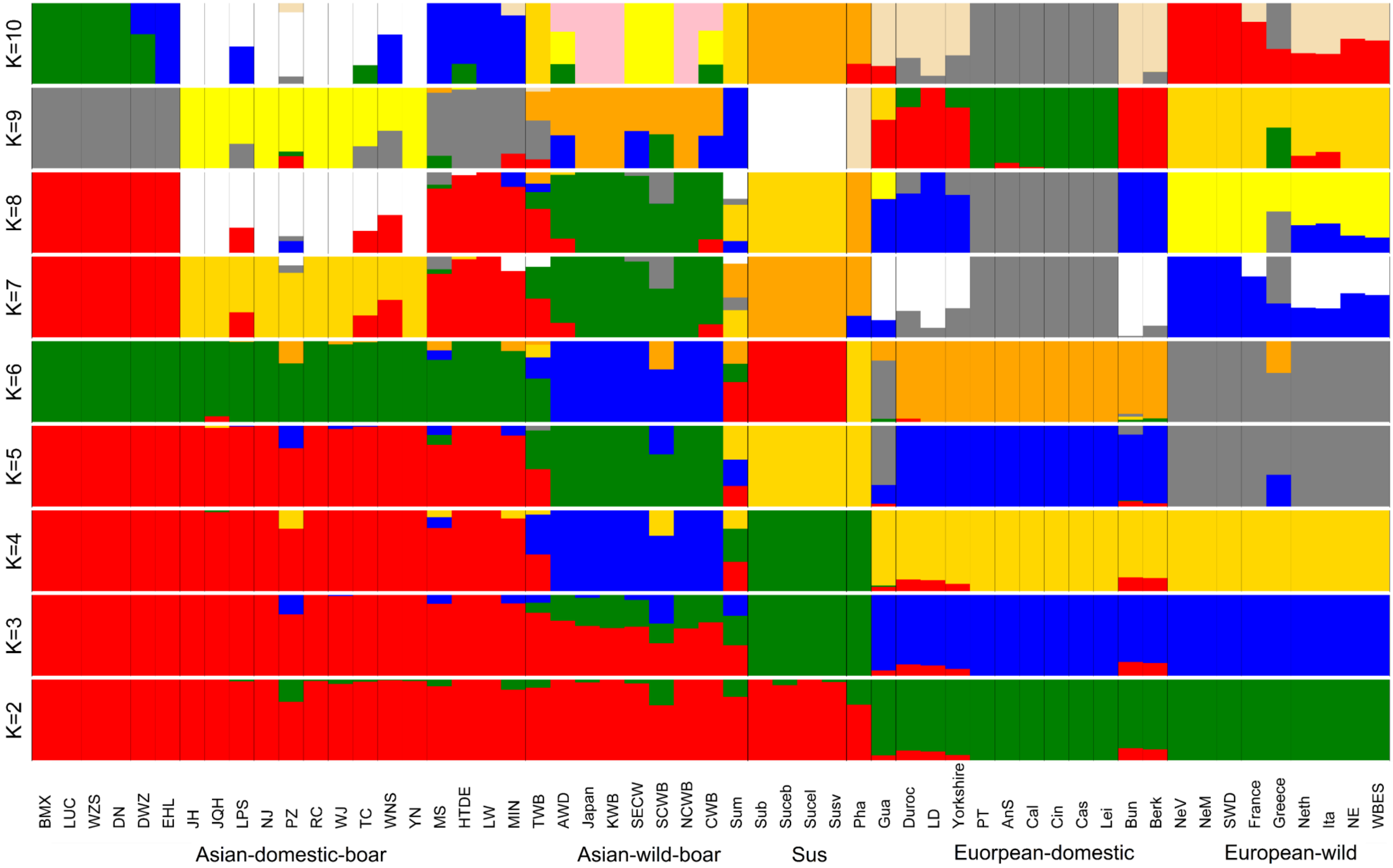

Figure S9 Admixture results of SVs.

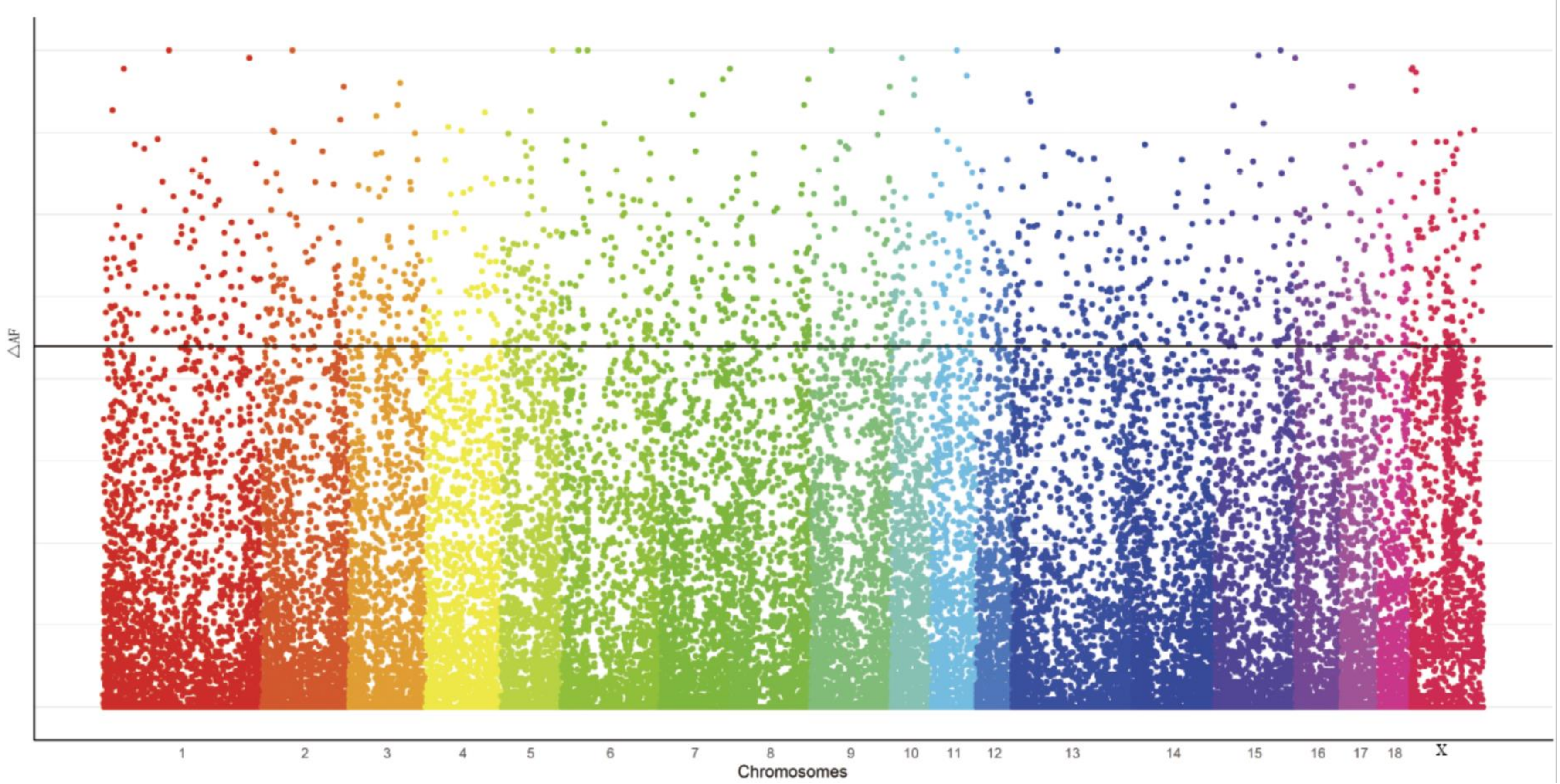

Figure S10 The plot of △AF values among Meishan, Duroc and Tibetan wild boar populations.

Reference

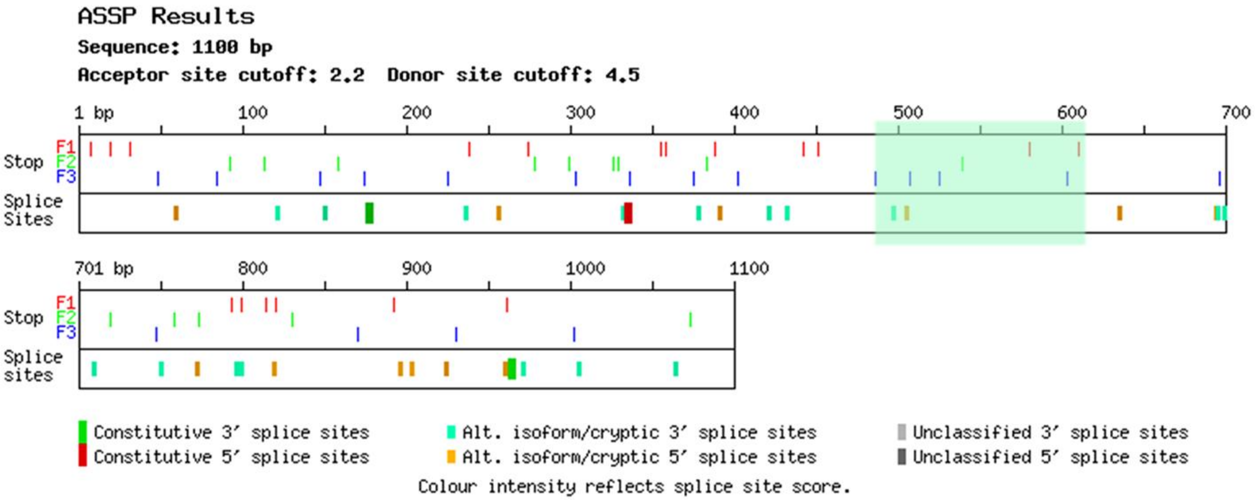

Meishan

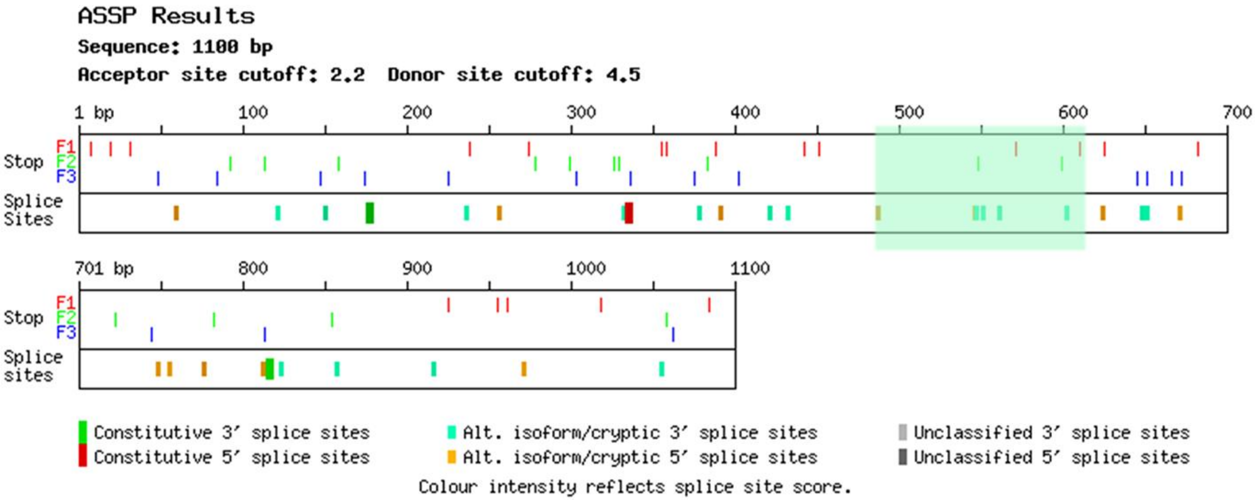

Figure S11 Alternative splicing prediction pf *IDO2*.

CYP2J2

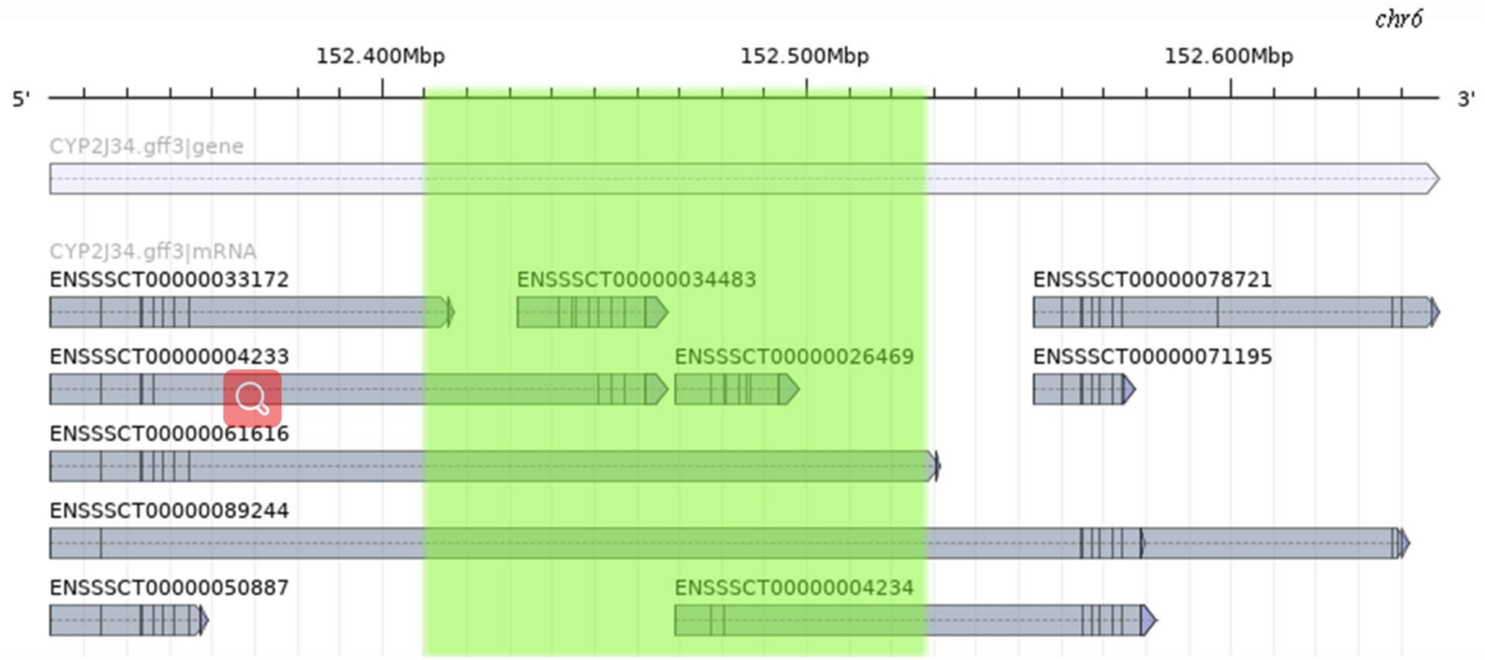

PLA2G4A

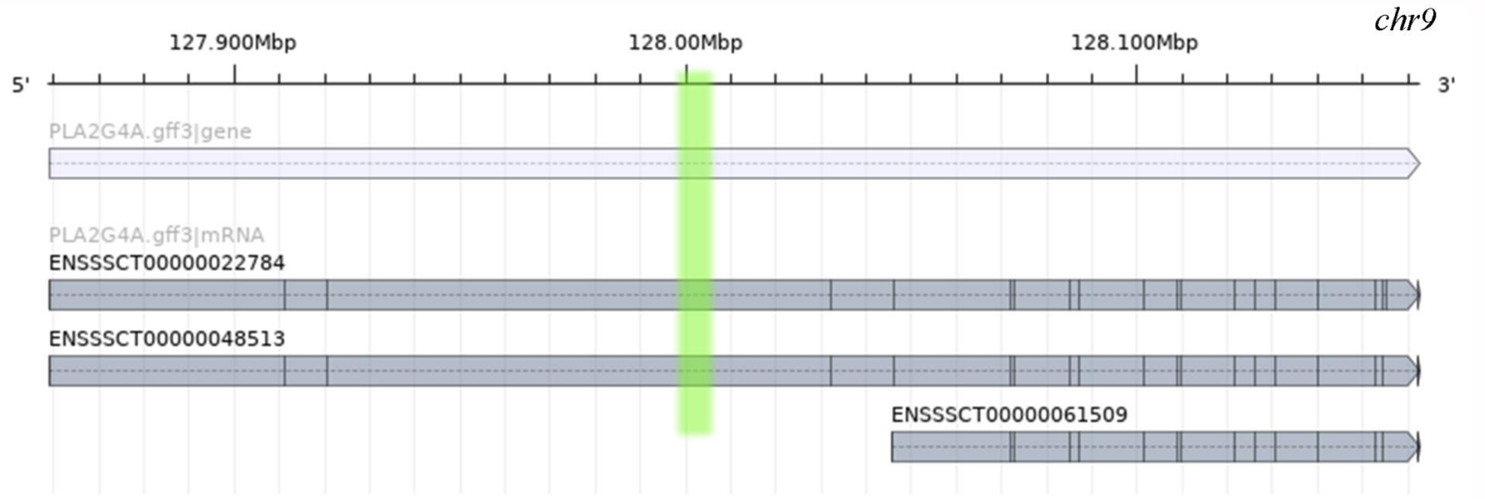

Figure S12 The duplication in *CYP2J2* and *PLA2G4A* gene.

The light green means the duplication regions.

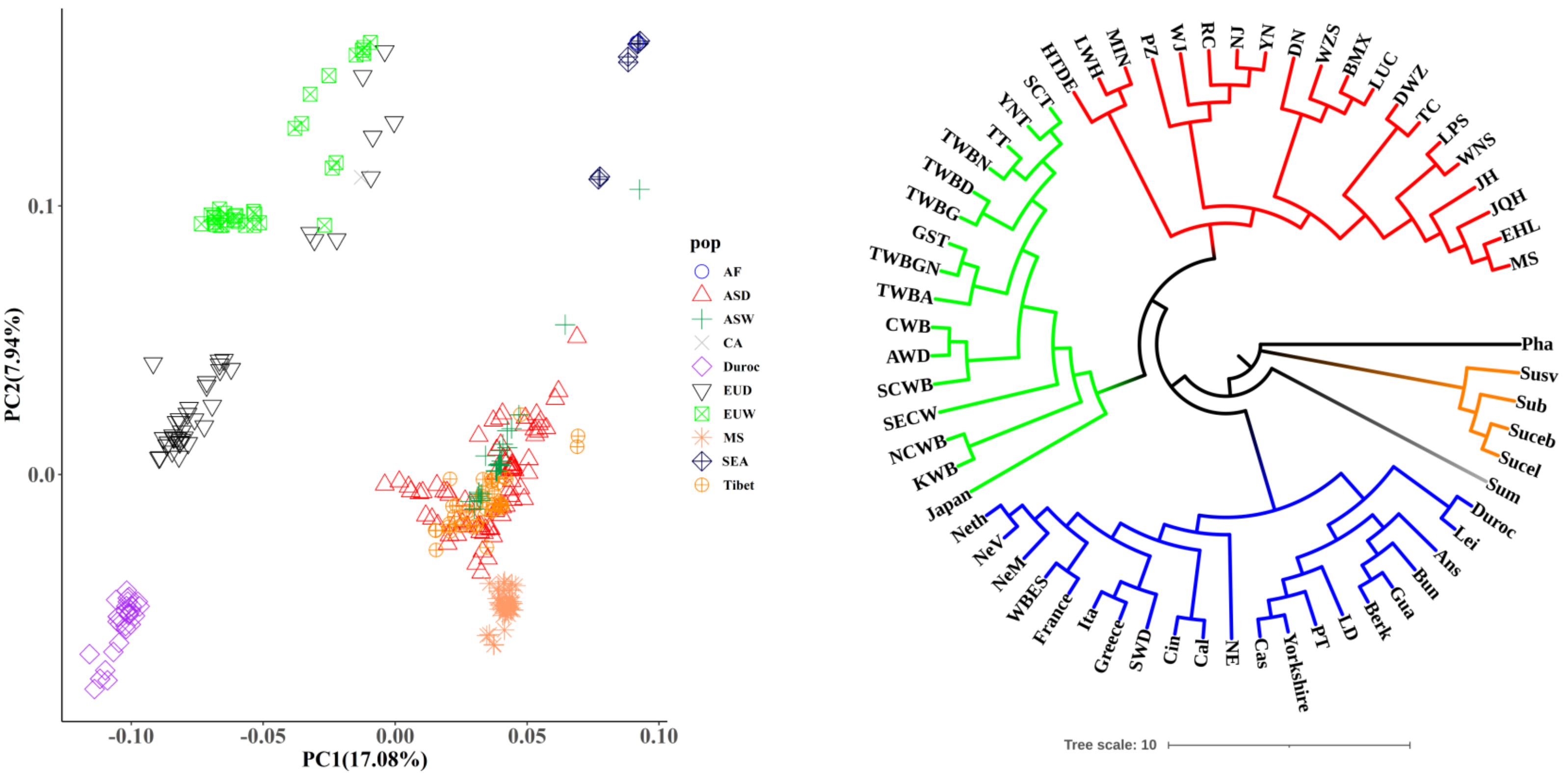

Figure S13 The PCA and NJ-tree of neutral “SNPs+SVs”.

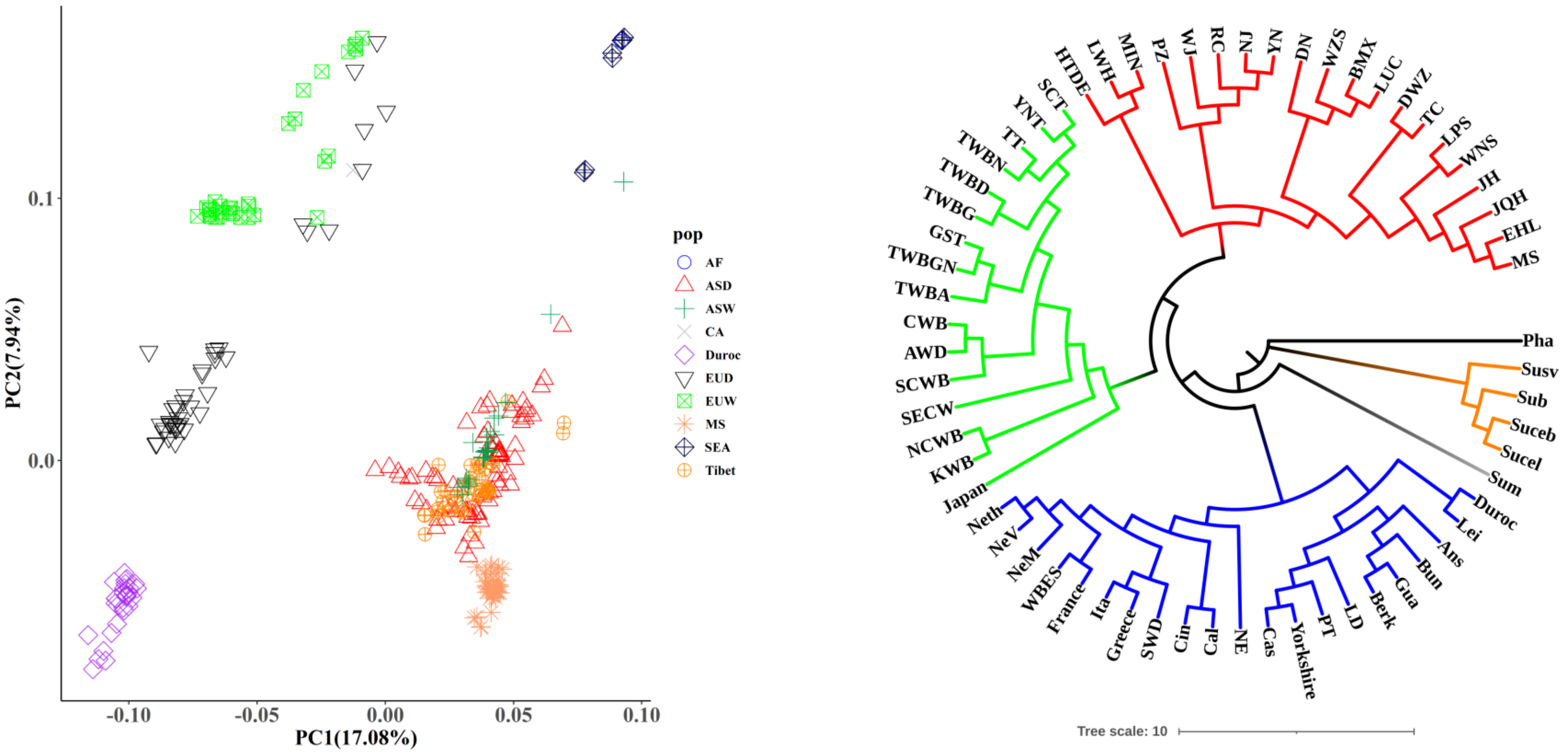

Figure S14 The PCA and NJ-tree of neutral SNPs.

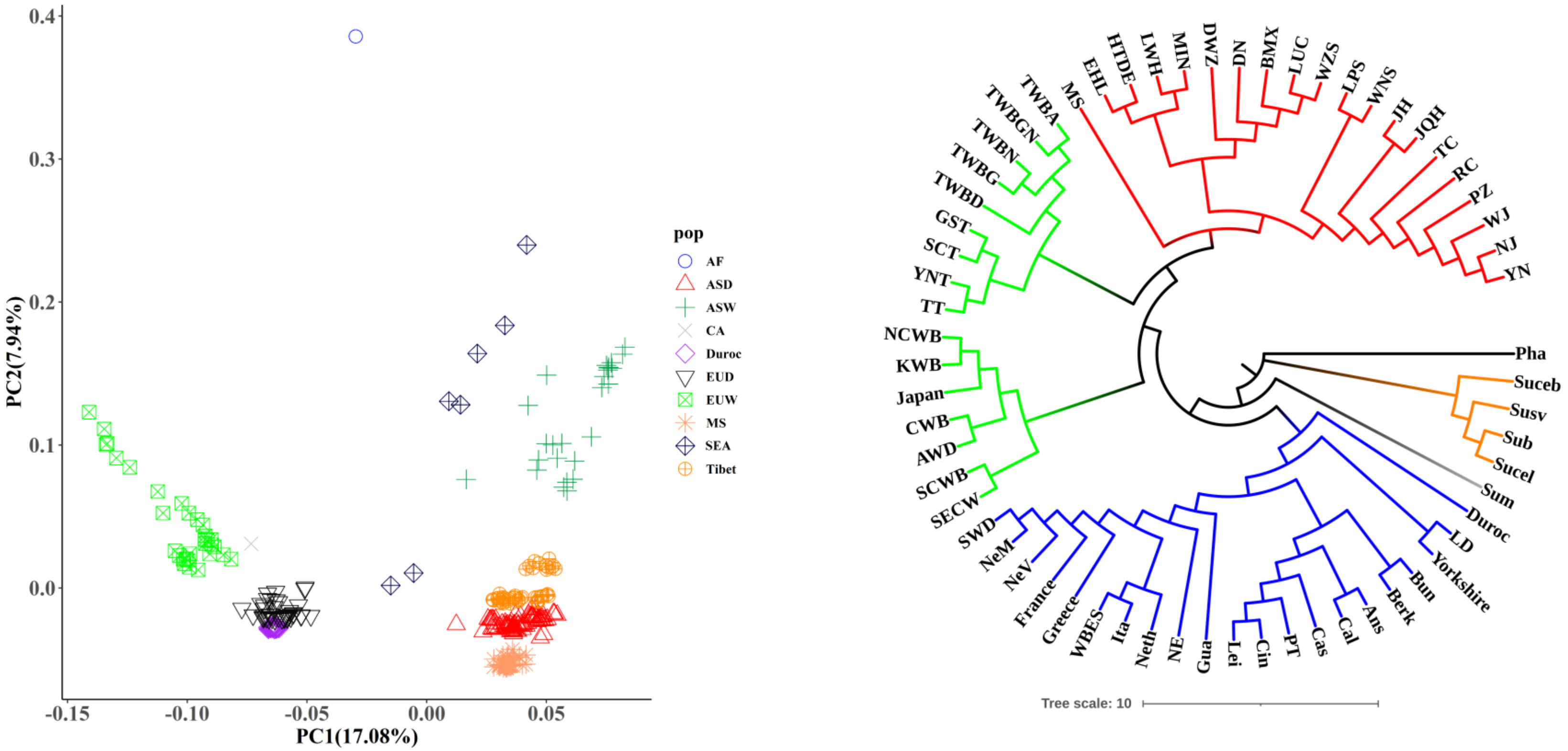

Figure S15 The PCA and NJ-tree of neutral SVs.

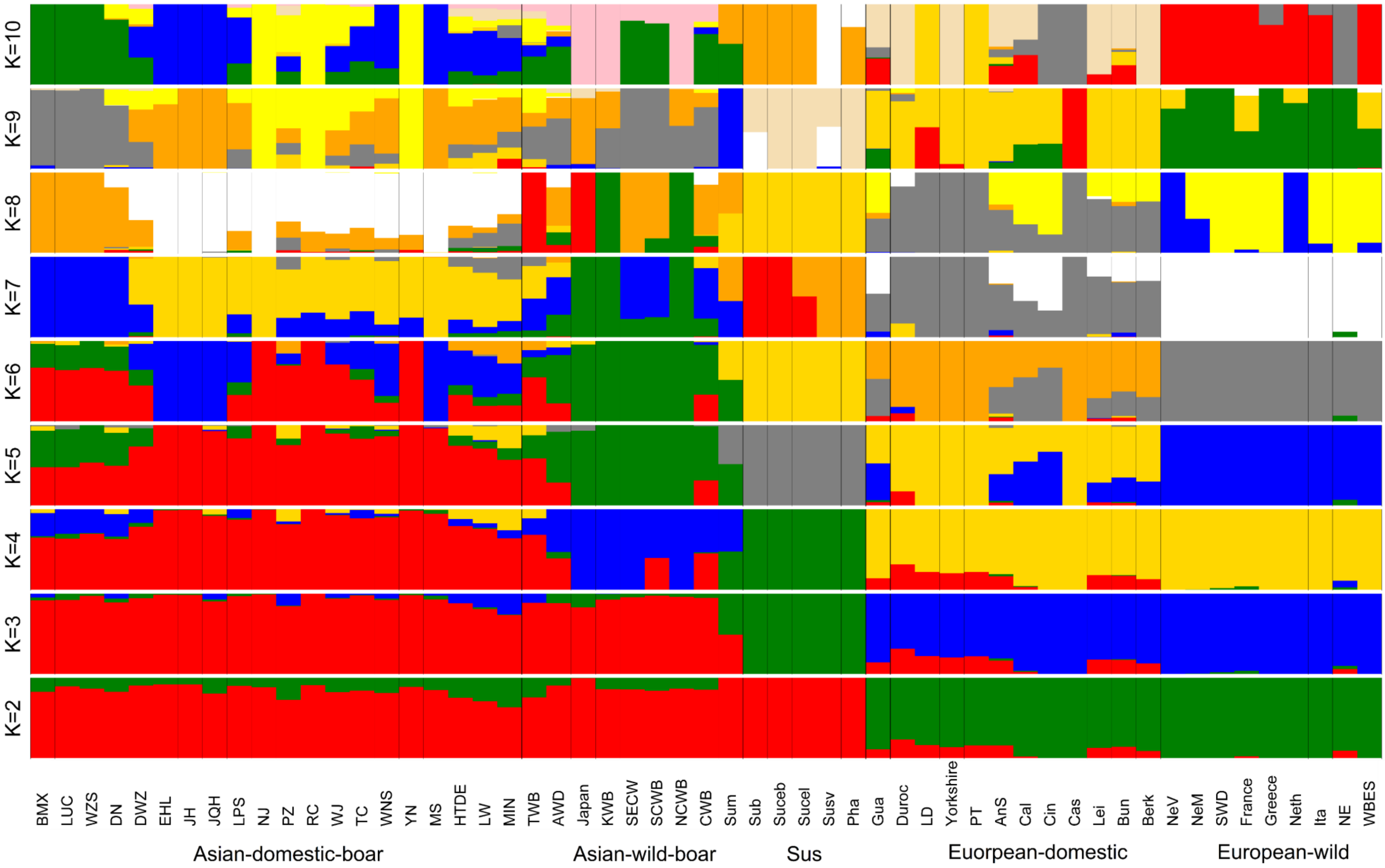

Figure S16 Admixture results of neutral “SNPs+SVs”.

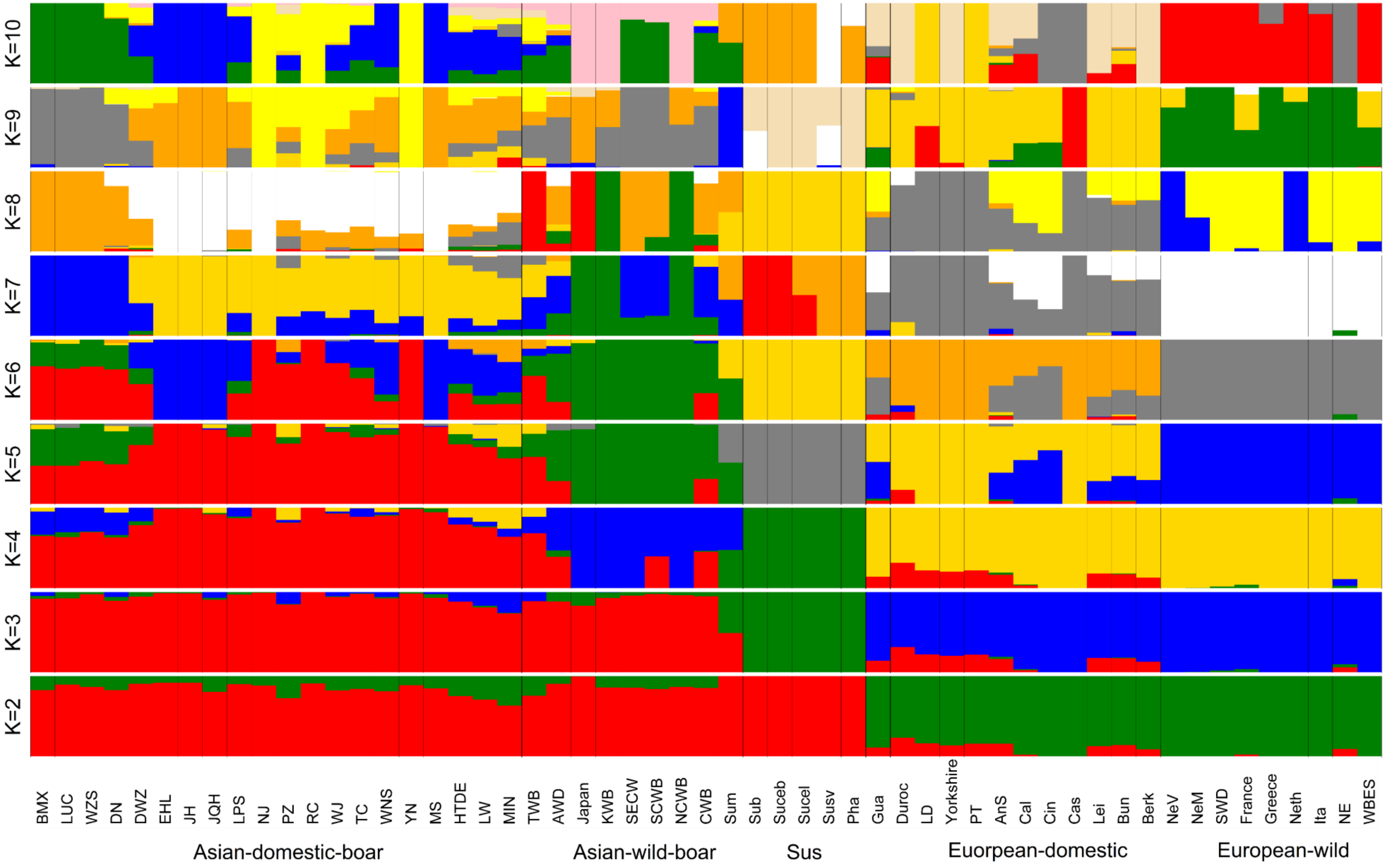

Figure S17 Admixture results of neutral SNPs.

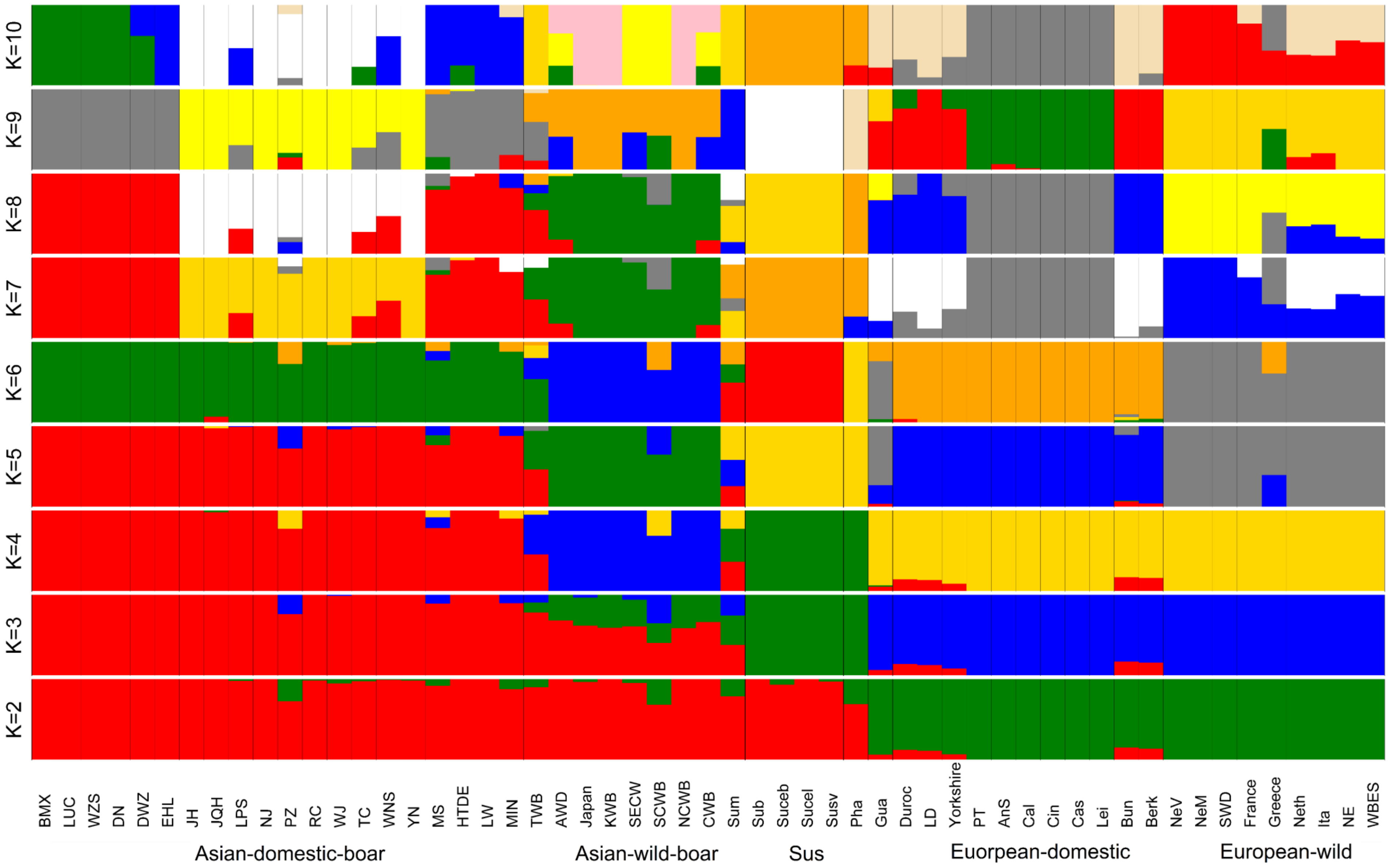

Figure S18 Admixture results of neutral SVs.
