# Supplementary material for: Analysis of Structural Variants Reveal Novel Selective Regions in the Genome of Meishan Pigs by Whole Genome Sequencing
Source: Front Genet. 2021 Feb 4;12:550676. doi: 10.3389/fgene.2021.550676 (PMC7890942; doi:10.3389/fgene.2021.550676)
Supplement: Supplementary file 3 [file Presentation_1.pdf]

1. Reads alignment

Reads were first quality controlled (parameter: -l 70 -s 20) and trimmed (parameter: -q 20) by NGSQC Toolkit v2.3.3 (Patel and Jain, 2012). Simultaneously, NGSQC Toolkit was also used to remove adapters. The filtered reads were aligned to the *S. scrofa* 11.1 reference genome using BWA v 0.7.17 (Li and Durbin, 2009). SAMtools v 1.9 (Li et al., 2009) and Picard v1.119 were used to reorder and sort alignment results. Genome Analysis Toolkit (GATK) v 3.8 (DePristo et al., 2011) was used to correct each individual's alignment results with marking duplicates, local realigning around indels and base quality score recalibration procedures.

2. SV detection

2.1 Methods

Six software were used to detect SVs, including Breakdancer, Pindel, DELLY, Manta, Genomestrip, and CNVnator.

Breakdancer was used to identify deletions (>100bp), insertions (>100bp), inversions, intra-chromosomal, and inter-chromosomal translocations according to the Read-pair algorithm(Chen et al., 2009). Pindel was utilized to detect short insertions and deletions, tandem duplications and inversions based on the Split-reads methods (Ye et al., 2009). DELLY and Manta were appropriated to discover size-extensive unbalanced rearrangements as well as balanced rearrangements. Furthermore, these two software all used Read-pair and Split-reads methods (Rausch et al., 2012; Chen et al., 2016). Genomestrip, integrating Read-pair, Read-depth and Split-reads algorithm, had the highly accurate and sensitive ability for deletions detection(Handsaker et al., 2015). Finally, CNVnator was used to investigate the copy number variations on the whole genome by the Read-depth method (Abyzov et al., 2011).

Because some low coverage samples were collected in our study, to ensure our SV detection accuracy, we first pre-filtered the results of all software. For the results of DELLY, Manta, Genomestrip and CNVnator, we removed the sites which read depth smaller than 8×. Because the average coverage of low-coverage samples (these samples' coverage smaller than 10×) was 6×, we choose 8× as the thresholds (average of 6 and 10) to filter the false positive sites and keep sites with more supporting reads. The site, which frequency was smaller than 0.05, was removed from the results of Breakdancer and Pindel.

After pre-filter, we merged the results of each software as follows:

(a) The two locus (except translocations), which reciprocal overlap >75%, exist in different software were considered as one locus (Sudmant et al., 2015).

(b) The SVs sites were retained only when two or more software simultaneously detected the site.

(c) The start and end of SVs were determined by calculating the median method. This method first collected all sites that were considered as the same SV. Then, the median of the start or end positions of all collection sites were calculated and regarded as the merged SV’s start or end positions, respectively.

(d) DELLY, Manta and Genomestrip can predict the genotype of each SV site. The genotype of one SV should be confirmed by the above two software, at least. Other sites and the sites detected by Pindel and Breakdancer were predicted by SVTyper (Chiang et al., 2015).

2.2 Results

The results of each software listed below:

	Software					
SV_type	Breakdancer	DELLY	Manta	Genomestrip	Pindel	CNVnator
Deletion	32097	55109	47680	31967	16402	
Insertion	14180	2865	3672		3153	
Duplication		9655	10087		5894	
Inversion	4541	18564	7608		12013	
Translocation	2126	0	134			
CNV						2040

3. SNP discovery

GATK and SAMtools were combined to call SNPs. The Haplotypecaller pipeline of GATK was used to detect and genotype variants. The results of SAMtools were supplied to select the reliable SNP sites of the GATK’s results using the “SelectVariants” function of GATK. After that, the SNPs were filtered, which not achieved the following requirements:

1) variant confidence/quality by depth > 2;

2) RMS mapping quality (MQ) > 40.0;

3) Phred-scaled P-value using Fisher’s exact test to detect strand bias < 60;

4) Z-score from the Wilcoxon rank sum test of Alt vs. Ref read MQs (MQRankSum) > −12.5;

5)Z-score from the Wilcoxon rank sum test of Alt vs. Ref read position bias (ReadPosRankSum) > −8;

Simultaneously, the site which read depth smaller than eight was removed. Finally, 18,358,356 SNPs were detected in 55 breeds.

4. Population genetic structure analysis

To use SVs to infer the pigs' population structure, we first extracted the genotype inferred by three software (Delly, Manta, and Genomestrip). The genotype inferred by at least two software was considered to represent the genotype of a site. All SVs in the SV call set were then genotyped using SVTyper v0.1.4 (Chiang et al., 2015).

Next, SNPs combined with deletions, deletions, and SNPs were separately used to estimate the relationships among all populations in our study. We then used these data to perform principal component analyses (PCA) (Yang et al., 2011). MEGA-X (Kumar et al., 2018) was used to construct the phylogenetic tree based on identity-by-state (IBD) matrices of the three datasets, respectively. The IBD matrices were calculated by PLINK v1.7(Purcell et al., 2007) using the parameters "--cluster --distance-matrix". The population structure analysis was conducted by Admixture v1.3 (Alexander et al., 2009). We also estimated the genetic background for each population. Nine possible groupings (K=2 to K=10) were calculated by Admixture, and the results were plotted using our R scripts.

We also compared the results of population structure analysis using variants before and after filtered non-neutral sites. The SNPs which filtered out non-synonymous variants were regarded as non-neutral sites. We did not find any significant differences between the results of using all variants and neutral variants. The results of population structure analysis using neutral variants were described in Figure S12-17.

Reference

Abyzov, A., Urban, A.E., Snyder, M., and Gerstein, M. (2011). CNVnator: An approach to discover, genotype, and characterize typical and atypical CNVs from family and population genome sequencing. *Genome Research* 21(6), 974-984.

Alexander, D.H., Novembre, J., and Lange, K. (2009). Fast model-based estimation of ancestry in unrelated individuals. *Genome Research* 19(9), 1655-1664. doi: 10.1101/gr.094052.109.

Chen, K., Wallis, J.W., McLellan, M.D., Larson, D.E., Kalicki, J.M., Pohl, C.S., et al. (2009). BreakDancer: an algorithm for high-resolution mapping of genomic structural variation. *Nature Methods* 6(9), 677-U676.

Chen, X.Y., Schulz-Trieglaff, O., Shaw, R., Barnes, B., Schlesinger, F., Kallberg, M., et al. (2016). Manta: rapid detection of structural variants and indels for germline and cancer sequencing applications. *Bioinformatics* 32(8), 1220-1222.

Chiang, C., Layer, R.M., Faust, G.G., Lindberg, M.R., Rose, D.B., Garrison, E.P., et al. (2015). SpeedSeq: ultra-fast personal genome analysis and interpretation. *Nature Methods* 12(10), 966-968.

DePristo, M.A., Banks, E., Poplin, R., Garimella, K.V., Maguire, J.R., Hartl, C., et al. (2011). A framework for variation discovery and genotyping using next-generation DNA sequencing data. *Nature*

Genetics 43(5), 491-+. doi: 10.1038/ng.806.

Handsaker, R.E., Van Doren, V., Berman, J.R., Genovese, G., Kashin, S., Boettger, L.M., et al. (2015). Large multiallelic copy number variations in humans. *Nature Genetics* 47(3), 296-+.

Kumar, S., Stecher, G., Li, M., Knyaz, C., and Tamura, K. (2018). MEGA X: Molecular Evolutionary Genetics Analysis across Computing Platforms. *Molecular Biology and Evolution* 35(6), 1547-1549. doi: 10.1093/molbey/msy096.

Li, H., and Durbin, R. (2009). Fast and accurate short read alignment with Burrows-Wheeler transform. *Bioinformatics* 25(14), 1754-1760. doi: 10.1093/bioinformatics/btp324.

Li, H., Handsaker, B., Wysoker, A., Fennell, T., Ruan, J., Homer, N., et al. (2009). The Sequence Alignment/Map format and SAMtools. *Bioinformatics* 25(16), 2078-2079. doi: 10.1093/bioinformatics/btp352.

Patel, R.K., and Jain, M. (2012). NGS QC Toolkit: A Toolkit for Quality Control of Next Generation Sequencing Data. *Plos One* 7(2). doi: ARTN e30619

10.1371/journal.pone.0030619.

Purcell, S., Neale, B., Todd-Brown, K., Thomas, L., Ferreira, M.A.R., Bender, D., et al. (2007). PLINK: A tool set for whole-genome association and population-based linkage analyses. *American Journal of Human Genetics* 81(3), 559-575. doi: 10.1086/519795.

Rausch, T., Zichner, T., Schlattl, A., Stutz, A.M., Benes, V., and Korbel, J.O. (2012). DELLY: structural variant discovery by integrated paired-end and split-read analysis. *Bioinformatics* 28(18), i333-i339. doi: 10.1093/bioinformatics/bts378.

Sudmant, P.H., Rausch, T., Gardner, E.J., Handsaker, R.E., Abyzov, A., Huddleston, J., et al. (2015). An integrated map of structural variation in 2,504 human genomes. *Nature* 526(7571), 75-+.

Yang, J.A., Lee, S.H., Goddard, M.E., and Visscher, P.M. (2011). GCTA: A Tool for Genome-wide Complex Trait Analysis. *American Journal of Human Genetics* 88(1), 76-82. doi: 10.1016/j.ajhg.2010.11.011.

Ye, K., Schulz, M.H., Long, Q., Apweiler, R., and Ning, Z.M. (2009). Pindel: a pattern growth approach to detect break points of large deletions and medium sized insertions from paired-end short reads. *Bioinformatics* 25(21), 2865-2871.
